# Opioid use disorder treatment disruptions during the early COVID-19 pandemic and other emergent disasters: a scoping review addressing dual public health emergencies

**DOI:** 10.1186/s12889-021-11495-0

**Published:** 2021-07-28

**Authors:** Rita Henderson, Ashley McInnes, Leslee Mackey, Myles Bruised Head, Lindsay Crowshoe, Jessica Hann, Jake Hayward, Brian R. Holroyd, Eddy Lang, Bonnie Larson, Ashley Jane Leonard, Steven Persaud, Khalil Raghavji, Chris Sarin, Hakique Virani, Iskotoahka William Wadsworth, Stacey Whitman, Patrick McLane

**Affiliations:** 1 Department of Family Medicine, University of Calgary, 2500 University Drive NW, Calgary, AB T2N 1N4 Canada; 2 Department of Emergency Medicine, University of Alberta, 8440 112 St. NW, Edmonton, AB T6G 2R7 Canada; 3 Blood Tribe, Stand Off, Canada; 4 Addiction Recovery and Community Health (ARCH) Team, Royal Alexandra Hospital, 10240 Kingsway NW, Edmonton, AB T5H 3V9 Canada; 5 Emergency Strategic Clinical Network, Alberta Health Services, 10030 – 107 St NW, Edmonton, AB T5J 3E4 Canada; 6 Department of Emergency Medicine, University of Calgary, 3330 Hospital Dr NW, Calgary, AB T2N 4N1 Canada; 7 Knowledge Resource Service, Alberta Health Services, 10030 – 107 St NW, Edmonton, AB T5J 3E4 Canada; 8 Metro City Medical Clinic, 150 - 909 5 Ave SW, Calgary, AB T2P 3G5 Canada; 9 Indigenous Services Canada, Suite 730, 9700 Jasper Avenue, Edmonton, AB T5J 4C3 Canada; 10 Department of Community Health Sciences, University of Calgary, 2500 University Drive NW, Calgary, AB T2N 1N4 Canada; 11 Department of Medicine, University of Alberta, 8440 112 St. NW, Edmonton, AB T6G 2R7 Canada; 12 Treaty 7 Chiefs Alliance, 206 - 8408 Elbow Drive SW, Calgary, Alberta T2V 1K7 Canada; 13 Addiction and Mental Health, Alberta Health Services, 10030 – 107 St NW, Edmonton, AB T5J 3E4 Canada

**Keywords:** Coronavirus, Disaster planning, Opioid epidemic, Emergency, Health services, Opioid agonist treatment, Medication assisted treatment, Review

## Abstract

**Background:**

During public health emergencies, people with opioid use disorder (PWOUD) may be particularly impacted. Emergent disasters such as the COVID-19 pandemic disrupt already-strained harm reduction efforts and treatment availability. This study aims to answer three research questions. How do public health emergencies impact PWOUD? How can health systems respond to novel public health emergencies to serve PWOUD? How can the results of this scoping review be contextualized to the province of Alberta to inform local stakeholder responses to the pandemic?

**Methods:**

We conducted a scoping review using the 6-stage Arksey and O’Malley framework to analyse early-pandemic and pre-pandemic disaster literature. The results of the scoping review were contextualized to the local pandemic response, through a Nominal Group Technique (NGT) process with frontline providers and stakeholders in Alberta, Canada.

**Results:**

Sixty one scientific journal articles and 72 grey literature resources were included after full-text screening. Forty sources pertained to early COVID-19 responses, and 21 focused on OUD treatment during other disasters. PWOUD may be more impacted than the general population by common COVID-19 stressors including loss of income, isolation, lack of rewarding activities, housing instability, as well as fear and anxiety. They may also face unique challenges including threats to drug supplies, stigma, difficulty accessing clean substance use supplies, and closure of substance use treatment centres. All of these impacts put PWOUD at risk of negative outcomes including fatal overdose. Two NGT groups were held. One group (*n* = 7) represented voices from urban services, and the other (*n* = 4) Indigenous contexts. Stakeholders suggested that simultaneous attention to multiple crises, with adequate resources to allow attention to both social and health systems issues, can prepare a system to serve PWOUD during disasters.

**Conclusion:**

This scoping review and NGT study uncovers how disasters impact PWOUD and offers suggestions for better serving PWOUD.

**Supplementary Information:**

The online version contains supplementary material available at 10.1186/s12889-021-11495-0.

## Background

When COVID-19 was declared a global public health emergency by the World Health Organization in March 2020, the opioid crisis was already impacting communities across Canada. Early public health documents highlighted that PWOUD may have more severe outcomes if infected with COVID-19, due to poorer baseline health and increased risk of transmission due to social inequities [1]. COVID-19 physical distancing measures also disrupt usual care and create new challenges for providers and public health decision-makers. Opioid Agonist Treatment (OAT) is the recommended treatment for OUD and increased morbidity and mortality are observed when OAT is interrupted [2]. In Canada, a surge of overdose-related deaths occurred early in the pandemic, as individuals became more likely to use drugs alone, with less access to services and supports to mitigate harms [3]. This study aims to answer three research questions. How do public health emergencies impact PWOUD? How can health systems quickly respond to novel public health emergencies to serve PWOUD? How can the results of this scoping review be contextualized to the province of Alberta to inform local stakeholder responses to the pandemic?

## Methods

We conducted a scoping review using Arksey and O’Malley’s six stage scoping review methodology [4] to: i) identify research questions; ii) identify relevant sources; iii) select sources; iv) chart data; v) collect, summarize; and report results; and vi) consult stakeholders.

### Identifying research questions

We identified our research questions in partnership with operational leaders and service providers in emergency and addiction care in the context of rising opioid deaths in Alberta early in the COVID-19 pandemic. In addition to our scholarly, knowledge-generating purpose, we had the knowledge translating purpose of contextualizing findings to Alberta. Following scoping review methods, our questions were refined throughout the course of the study as we gained familiarity with the literature, until they reached the final form reported in our introduction.

### Identify and select sources

To identify scientific literature, a librarian (AL) and research assistant searched 10 electronic databases in May/June 2020 with search terms related to: disease outbreaks or disasters; opioid and substance use disorder; health care services and access (Table 1; full search strategy available in Additional file 1).

**Table 1:** Scientific literature search strategy

Databases searched	Search terms
Ovid Medline, APA PsycINFO, CINAHL Complete, LitCOVID, WHO COVID-19 database, TripPro, Science Direct (which included searches in Science Direct Covid − 19 Research database & Elsevier 1Science Coronavirus Research Repository), Embase, Web of Science, and Ovid Cochrane Database of Systematic Reviews	COVID-19, Coronavirus, MERS-CoV, Middle East Respiratory Syndrome Coronavirus, Severe Acute Respiratory Syndrome, disease outbreak, influenza, opioid, opioid use disorder, substance use disorder, disaster, natural disaster or mass disaster, health care access, community mental health service, primary health care, community care, telehealth, health care disparity

**Fig. 1:**
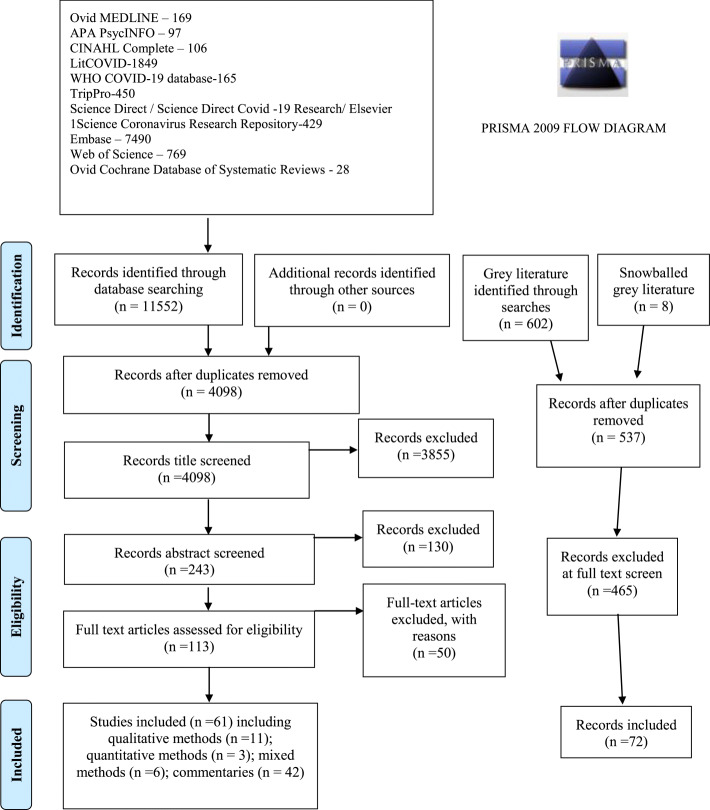
Literature search and study selection

Duplicates were removed and results screened for inclusion criteria through title, abstract, and full text review (Fig. 1). Final inclusion criteria were studies that: (i) were published in a scientific journal from 2000 to 2020; (ii) provided insight on PWOUD; (iii) informed changes to service delivery, care and access to treatment; (iv) examined a natural disaster, pandemic or crisis situation; and (v) had full text available. Since English search terms were used, only English results were identified.

To gather grey literature (i.e., non-academic sources), a librarian (AL) completed Google searches using six search strings in June 2020, limiting results to the COVID-19 pandemic, Canadian sources, and the top 100 results of each search string for manageability (see Additional file 1). Websites identified by the study team were also searched for key words (e.g., “COVID-19” OR coronavirus AND opioids OR “opioid use disorder” OR “substance use disorder”). Grey literature inclusion criteria were: (i) Canadian source; (ii) related to opioid use, services and supports; and (iii) specific to COVID-19. We limited our inclusion criteria for grey literature to Canadian sources as a matter of manageability, and given stakeholder interest in locally-applicable contemporary information.

Multiple team members were involved in each step of title and abstract screening, as well as full text screening, to elaborate upon and achieve consensus on inclusion criteria and application of these criteria. As per scoping review methods, the reason for having multiple team members involved in screening was to enhance understanding of the literature and its relevance to the research questions [4].

### Charting data

Tools for data extraction were developed and revised under the supervision of the first and last authors. We extracted source aims and methodology (when applicable), country, health service, disaster examined, disaster impacts (including affected populations and service disruptions), service adaptations, as well as resource type for grey literature (e.g. policy document, practice guideline, news article). Three reviewers were involved in data extraction, with 100% of scientific literature and 20% of grey literature extraction cross-checked by a second reviewer to enhance relevance and comprehensiveness of extracted information.

### Collecting, summarizing and reporting results

Literature review results were thematically outlined in information sheets in which analysts recorded common issues and ideas that appeared within sources, arranging these in bullet points with citations to relevant sources. Draft information sheets formed the basis for team discussion and organization of bullets under thematic headings. The information sheets formed an interim stage in analysis and were provided to stakeholders during Nominal Group Technique (NGT) sessions (discussed below) as a basis for co-interpretation of findings. Key themes presented to the stakeholders were: 1) increased risks during disasters for PWOUD, 2) models of care adaptations, and 3) cross-systems implications. Final tables of results, as they appear in this manuscript, were created based on stakeholder input. Our development of extraction forms, information sheets and final manuscript tables are in keeping with Levac, Colquhoun and O’Brien’s elaboration upon the Arksey and O’Malley framework, and their explanation that creation of data reporting products is part of the analysis work within qualitatively driven scoping reviews [5]. Overall, we adopted a generic qualitative approach involving reduction and display of data, in order to draw conclusions about relationships within the data and reach overarching themes [6, 7].

Reconsidering our results with stakeholders who work in the field of interest is an effort to enhance the rigour of our analysis through triangulation of perspectives, as well as a strategy for optimizing relevance of findings for health practitioners. Contextualizing scoping review results to the local pandemic response in Alberta is also a key component of this project, as universal or generalized approaches to care are not effective across diverse settings and populations.

### Consulting stakeholders

A NGT was selected for its collaborative approach to building consensus between diverse, multi-sectoral knowledge users, for its attention to context, and as an effective knowledge mobilization method, as the engaged stakeholders can apply the evidence within their spheres of influence. This approach supports a broad view of the healthcare system integrated with Indigenous ways of knowing, including attention to community, relationships and healing the whole person (see [8]). The NGT is also valuable for work on stigmatized behaviours such as substance use, as the focus on building consensus helps to mitigate the likelihood of divisive partisanship that could prevent reflection on systems issues. NGT attendees are co-investigators and co-interpreters of the data, rather than traditional study participants, and so did not sign ethics consent forms. Not all participants in the NGT met authorship criteria at final submission, and not all co-authors attended NGT sessions.

NGT groups were composed of care providers, systems-level decision-makers, and patient advocates from Alberta, Canada, as well as representation from First Nations stakeholders. Stakeholders were invited to identify where the literature was reflective of their own experiences in service settings during the early days of the COVID-19 pandemic and where the literature could better inform and support their practices. They contributed ideas and engaged in moderated discussions to prioritize core insights about the literature data and its limitations [9]. Notes were taken during NGTs by multiple team members and core insights were generated based on these notes. NGT stakeholders reviewed, revised and validated core insights presented in tables and texts.

## Results

After full text screening, 61 scientific journal sources met inclusion criteria, though these were primarily commentaries (*n* = 42, 60%) (Table 2). Peer-reviewed literature included 11 qualitative, three quantitative, and five mixed methods sources (*n* = 19). Forty sources pertained to early COVID-19 responses, 12 focused on OUD treatment during hurricanes and nine focused on OUD treatment during other disasters (e.g., 9/11, heatwaves, riots, earthquakes and disasters in general). We also synthesize COVID-specific scientific and grey literature below. Seventy-two grey literature results met inclusion criteria (See Additional file 2 for a full list of included scientific sources; Additional file 3 for a grey literature summary).

**Table 2:** Scientific source overview

	COVID-19 Focus	Other Disaster	Total
**Methodology**			
Qualitative	1	10	11
Quantitative	0	3	3
Mixed Methods	0	5	5
Commentary	39	3	42
**Region**			
United States	24	15	39
Global	7	1	8
Canada	1	0	1
Other	8	5	13
**Health System**			
General	13	6	19
Specialty or Addiction-focused	20	11	31
Cross-Systems	3	1	4
Other	4	3	7
**Total**	40	21	61

### Literature review results - impacts of disasters on PWOUD

The literature reports that PWOUD may be more impacted than the general population by COVID-19 stressors, including loss of income, isolation, lack of rewarding activities, housing instability, as well as fear and anxiety [10, 11] (See Additional file 4 for a literature summary sheet). They may also face unique challenges including threats to drug supplies, stigma, difficulty accessing clean substance use supplies (e.g. needles) and closure of substance use treatment centres) [1, 10, 12] . Stress is likely to worsen substance use issues and increase high risk or undesired use of substances [11, 13–15]. This may be acutely felt by those accessing OAT or who consider themselves to be in recovery, particularly for low income and marginalized groups [10]. Scientific literature indicated early on that physical distancing causes isolation and lack of rewarding activities, possible risk factors for increased substance use, self-harm, domestic violence, and other mental health problems [11]. Stressors can lead to substance use disorder development, intensification of substance use, or renewed high risk or undesired use of substances for those whose OUD was stably managed through treatment [13–15]. PWOUD may also experience increased difficulty obtaining sufficient supplies (e.g. food, substances and clean supplies for substance use) to shelter in place for extended periods, heightening risks [1]. For instance, needle shortages may result in reuse or sharing, and in turn transmission of bloodborne diseases such as HIV and Hepatitis C [1]. These stressors were also common themes in peer-reviewed sources on previous disasters (see Table 3).

**Table 3:** Summary of findings from peer-reviewed sources on previous disasters

Amplified Risk for PWOUD during Disasters	Efforts to Mitigate Risk for PWOUD and their Essential Services During and After Disasters
• Disasters create high-risk environments that exacerbate substance use and risk of infectious disease spread [16]. • After disasters, people who resume illicit drug use after a period of abstinence or use of safer supply do so in a higher risk context. Decreased purity of illicit supply has been noted after disasters and fears of scarce supply can result in high risk behaviour like sharing of needles [16, 17]. • Personal impacts such as decreased employment, difficulty accessing basic needs, homelessness, lack of transportation, lack of information on how to access OAT and other supports, discrimination and stigma may result in the use of substances to cope with disaster contexts [16, 17]. • Systems issues such as decreases or redirection in public health spending towards disaster relief, disruption to substance use treatment and disruption to harm reduction services increase risks for PWOUD after disasters [18]. • During and after disasters, psychological and emotional distress increases for both PWOUD and staff of support programs who are also personally experiencing the disaster [19]. • Disruption of services after disasters and increase in homelessness associated with some disasters cause psychiatric distress and may increase substance use [18], and displaced populations that rely on shelters can be met with unprepared or untrained staff [20]. • Disruptions in OAT services, inadequate take home dosing, lack of guest-dosing information at alternate clinic sites put PWOUD at increased risk for negative outcomes after a disaster [19, 21]. • When OAT care is disrupted, people turn to emergency departments for access to OAT medications. However emergency clinicians sometimes face barriers prescribing OAT or lack access to patient dosing information, resulting in inadequate or unsafe prescriptions [21].	• Efforts to ensure access to OAT include: Provision of take home dosing, guest dosing at clinics other than the patients’ usual clinic, delivering/mailing of medication to patients, mobile units and communication strategies (e.g., individual phone calls, hotlines and social media) to keep people informed on how to access treatment [21, 22]. Other supports include: • Mental health support for fear & anxiety after disasters: lack of increase in illicit drug use attributed to availability of mental health professionals, support groups, and counsellors [23]. • Internet-based modules providing psychoeducation and motivational feedback focused on mental health and substance use issues after a disaster [24]. • Disaster planning that values cultural specificity and needs of people who have disabilities, mental health issues, use substances, or are on OAT to ensure providers, first responders, organizations, and emergency managers are prepared for disaster scenarios [22]. • Formal disaster plans and a central database containing dosing information [21, 22] and coordinated emergency laws [20].

Literature reported reduced access to addiction treatment, recovery supports, and harm reduction services, leading to increased health and safety risks for PWOUD [14]. Disruptions in OAT access can cause withdrawal symptoms, leading some to seek illicit supplies [25] and increasing the risk of overdose due to more toxic or new and unfamiliar products in circulation [14]. As well, periodic voluntary or involuntary abstinence also increases risk of overdose, and may have been more common early on during COVID-19 due to interruptions in treatment, efforts to shelter in place and changes in street-level drug supply [26]. Additionally, decreased access and availability of naloxone during the early COVID-19 period [15], and fears of COVID-19 transmission through nasal naloxone and due to a lack of personal protective equipment (PPE) may have resulted in less overdose rescues before PPE supplies stabilized and aerosol protocols were established [14].

COVID-19 also intensifies already-existing barriers to care for underserved populations [27], including through quick clinic closures in response to the pandemic, decreased access to supervised consumption sites (SCS), and increase of drug use in isolation [14]. Patients may experience increased difficulties navigating systems that are even less coordinated than before the pandemic, as attention focused on provider- and clinic-level emergent COVID-19 guidelines and protocols [28].

The correlate of increased risks and disruptions for PWOUD appeared in the literature through efforts to mitigate substance use risks during COVID-19. In some jurisdictions these included clinical guidelines for risk-mitigation opioid prescribing and for reducing the risk of COVID-19 transmission [29], or shifting to telehealth, smaller patient numbers in group therapy, and hand sanitizer provision [13, 30]. One source suggested telemedicine combined with street outreach as a holistic approach, noting that tailored care has been shown to improve housing stability and mental health along with care access [31]. At a policy level, Health Canada published exemptions to make OAT-prescribing more flexible and decrease in-person visits though virtual initiation of OAT, longer length of prescriptions, reduction of urine tests and witnessed dosing requirements, verbal prescription transfers to pharmacies closest to the patient, delivery of OAT by pharmacies, and allowing friends and family of patients to pick up OAT doses [32]. Similar shifts in care and the argument to maintain disaster-driven shifts as good practice, as well as the need for disaster planning, were common themes in peer-reviewed sources on previous disasters (see Table 3).

### NGT results

The first of the two NGT groups held (*n* = 7) represented voices from urban services, and the second (*n* = 4) represented voices from Indigenous contexts including First Nations reserve settings. Stakeholders elaborated upon ideas present within the literature with issues faced in local contexts, as presented in Table 4. Stakeholders also felt key issues that they faced during the first months of the COVID-19 pandemic were not adequately discussed in the literature. NGT stakeholders felt that the intersectionality of multiple stigmatized identities should be acknowledged, noting that the literature missed differential impacts of COVID-19 disruptions on Indigenous people, who are impacted by racist stereotypes that link Indigeneity to problematic substance use (see also [34]). Stakeholders also emphasized that early COVID-19 disruptions intensify adversities for people in precarious circumstances and increase risk-taking to meet basic needs. Such risk taking may involve participation in informal (often criminalized) economies including sex work. Providers expressed concern that increased overdoses were partially due to responder uncertainty about the risk of contracting COVID-19 during an overdose response, though guidance documents were available in some jurisdictions [29].

**Table 4:** Stakeholder Contextualization of Literature to Social Disruptions from COVID-19

SOCIAL CONTEXT OF DISRUPTION		
**What resonates from the literature?**		**Paraphrase of Stakeholder Comments**
***Social isolation***	• Greater substance use in isolation; scarcer spaces & disrupted networks to more safely use drugs in groups. • Increased illicit substance use from unfamiliar sources; drug supply shifts potentially increasing toxicity. • Amplified quality of life vulnerabilities for PWOUD; “relapse” part of broader substance use intensification. • Sudden income loss and difficulties to secure basic needs driving increased stress & risk taking.	*People coming out of incarceration or hospitals are finding their map of where to access normal services have changed, and many don’t know how to navigate not just what is available, but don’t have means* via *available transportation.*
**Where could the literature go further?**		**Paraphrase of Stakeholder Comments**
***Intensified adversities***	• Decreased overall support from social and health services due to closures for physical distancing and planning needed to prepare for a communicable disease pandemic. • Pandemic Income assistance disrupted eligibility for other social assistance, which sometimes led to loss of medication coverage and new barriers. • Disparate approaches to mitigate risk, with pandemic efforts emphasizing COVID-induced barriers to care without sufficient attention to pre-existing gaps in care. • Disruptions differentially impact racialized, gender minority, housing insecure, and other vulnerable groups in specific ways that need to be better understood and addressed.	*The crisis for people experiencing OUD is worse than COVID.* *We have to compare these two epidemics locally* (COVID & overdose)*, we must call it a dual public health emergency. It 100% affects all, just as infectious diseases do.* *Need to look disparity in the eye, why treat COVID with urgency and take away resources and increase risks elsewhere?*
***Stigma***	• Disruptions aggravate existing adversities & decrease access to care for already underserved groups. • While focus on stigma facing PWOUD is important, it may limit attention to intersectionality of multiple stigmatized identities, especially racial & gender inequities.	*It’s like Maslow’s hierarchy of needs*^*1*^*: when on treatment for addiction, you’re a bit tied to healthcare and there’s a razor’s edge of needs to satisfy at the same time, to eat and drink and stay alive in a toxic environment. We’re seeing the system not meet those needs and being politicized. For Indigenous PWOUD, you have 500 years of colonization, then this pandemic that isolates and incarcerates people for trying to meet basic needs.* *They’re not bad people but the stigma that they face … people are dying because of racism.*

See [33]

Stakeholders took issue with Alberta public health authorities’ perceived tendency to prepare for and respond to one crisis at a time, with limited capacity to tailor public health responses to the unique needs of PWOUD who will be predictably affected in unique ways by emergent disasters. For these stakeholders, system disconnection, the need for innovation, a dearth of up-to-date information and contextual guidance, and the need for public health to balance multiple crises at once, all converge in the need for systems and service accountability to PWOUD. Stakeholders reported there were many unknowns and very little support for community providers and pharmacies, with most of the initial resources directed to acute care. This was perceived to increase gaps in care, particularly for PWOUD who lack telephones or accessible transportation to sustain contact with their providers (e.g., pharmacists, physicians, social workers) during a disaster.

Providers emphasized that their regulated professional bodies require them to respond to the needs of their clientele and maintain high standards of practice. Yet they also outlined gaps in their ability to provide care without systems-level support. This undermines accountability to patients and to providers, who are susceptible to burnout without the resources necessary to support their patients.

Providers further noted that the neighboring province of British Columbia had early access to data and practice guidelines. They felt British Columbia seemed to engage in evidence-informed decision-making that took into account both social and health systems issues. Many providers reported turning to sources from British Columbia to guide their practice and understanding of the needs of PWOUD in their care. Stakeholders described uneven political responses within distinct jurisdictions, noting that health authorities in British Columbia increased capacity for risk mitigation opioid prescribing.

## Discussion: outcomes of COVID-19 disruptions

Emergent disasters increase burden on PWOUD trying to meet basic needs (such as shelter, food, substances, and healthcare), and aggravate risk behaviour by intensifying reliance on informal economies, and more frequent (and dangerous) substance use in isolation. Disaster literature pre-COVID-19 shows that the intensification of adversities faced by PWOUD during disasters is predictable. Public health has little reason not to anticipate the unique consequences of emergent disasters for medically underserved or socially vulnerable groups. Preparation for how disasters will impact vulnerable populations, including PWOUD, should involve nurturing relationships between providers that patients access across complex health and social services systems (e.g., establishing lines of contact, mandating coordinated care). As shown by our review of grey literature, early COVID-19 public health guidelines generally did not attend to the social realities of PWOUD. In future, public health should anticipate negative effects of public health measures and new hazards for populations at risk for catastrophic results of combined crises, rather than focusing attention on one crisis at a time.

Early public health responses to the pandemic identified COVID-19 as the primary threat to life, yet local outcomes raise questions about this assumption. An Alberta Health report on opioid deaths from Spring 2020 reported the highest ever number of opioid-related deaths in a single three-month period in Alberta [35]. In March 2020, OAT clinic operations were disrupted due to the pandemic [35]. SCS data indicates a fall in service uptake in Spring 2020 following capacity reduction measures in adherence with public health distancing guidelines [36].

While the COVID-19 death rate would almost certainly have been higher without the public health measures, avoiding COVID-19 deaths and preventing overdose deaths need not be understood as goals in opposition to one another. The dual pubic health crises could be equally addressed through evidence-informed measures that anticipate and address patient needs. This review highlights that systems that are more attentive to social determinants of health and that prioritize contextually-tailored care are better prepared for disruptions as they emerge.

COVID-19 adaptations to OAT access have focused on flexibility measures (e.g. take-home dosing, telehealth, mobile clinics) that may have helped many, but have largely relied on individual patient and provider adaptations, without systemic supports. This lack of system and service accountability to address emergent patient needs early during disruptive events is avoidable and puts unnecessary burdens on patients and providers. We present recommendations for system and service accountability in OUD care during disasters in Table 5.

**Table 5:** System & Service Accountability for Responsive OUD Care during Disaster-Driven Disruptions

Context of Disruption	Public Health Mechanisms to Mitigate Risks	Expected Outcomes
Disasters focus attention on single risks & generalized solutions	Prepare cross-systems protocols & coordinate to anticipate how disruptions affect populations rendered at risk.	Mitigate multiple sources of risk by attending to patients’ as whole persons & diverse populations in widely varying social contexts. Engage in theoretically and historically-informed planning to anticipate risk & project implementation to mitigate future risks. Avoid using emergency departments as universal safety nets during disasters.
	Anticipate, track, and address risks from emergent disasters as they interact with risks from associated social and health systems disruptions (e.g., impacts of pandemic as well as of distancing measures).	
	Orient health system data analytics to generate & circulate knowledge on multiple sources of risk and population groups.	
Lack of information transparency in decision-making perpetuates stigma & produces policy inattentive to social determinants	Address social determinants of population health inequities (including racism) by tailoring public health guidelines for socially vulnerable groups (e.g., feasible, accessible, effective measures).	Prevent misinformation and reduce stigma by grounding policy and service decisions in evidence around what drives increased risk from disasters (e.g., that disruption in financial situations of people in poverty increases negative outcomes)
	Enhance supports linking social & medical systems for vulnerable populations during disasters to prevent predictable intensification of adversities & treat addictions services equitably with other chronic/pre-existing diseases services that received additional tools and guidelines.	
Harm reduction & contextually-tailored care	Ensure safer supply of opioids and supplies to help PWOUD through an emergency, while helping them to access other components of care.	The system accommodates more change than individual patients are expected to accommodate. The burden of trying to determine what constitutes high quality care or appropriate attention to patient needs is not put on individual, unsupported, providers or care settings acting in isolation, and is instead achieved through a collaborative public health system.
	Empower systems & service providers; shift burden to the system to minimize strain on patients.	
	Support providers with informed order sets, care pathways, lists of resources, and links to social service and community partners to enable them to provide high quality and contextually-tailored care.	

Predicting the needs of diverse populations and their providers could prevent systems from becoming overwhelmed. Systems can be supported and funded to be more ready and less reactionary when the unexpected happens. Funded supports might include clinical and office space, as well as staff such as implementation leads, policy writers, planners, case managers, and social workers. Such funded supports can provide for informed approaches to both social realities and health systems issues, and examples of successful models exist. At the service level, health system navigation and case management for chronically ill and unstably housed patients has shown promise in addressing social determinants of health [37]. At the system level, the Emergency Strategic Clinical Network built referral pathways between emergency departments and addiction treatment clinics prior to the pandemic [38].

Such approaches, and the kinds of public health approaches requested by NGT stakeholders, are united by the concepts of harm reduction and contextually tailored care [39]. Hyshka and colleagues describe “ideal” harm reduction frameworks as reflecting 17 components “including a focus on preventing harm and not substance use per se, tailoring approaches to specific needs of populations, addressing underlying causes of drug-related harm, involving [persons who use drugs] in decision-making, [being] evidence-based, rights-orientated” and considering social determinants of health [40]. Ford-Gilboe and colleagues describe contextually-tailored care as an approach that “expands the individually focused concept of patient-centered care to include offering services tailored to the specific health care organization, the populations served, and the local and wider social contexts [39].” Taken together, harm reduction and contextually-tailored care approaches suggest going beyond a focus on single issue responses to crises, and recognition that PWOUD cannot safely have their treatment or substance use interrupted during emergent disasters.

### Limitations

The COVID-19 pandemic is now over a year old, and an important consideration for interpreting this review is our focus on novel public health disasters and early systems responses. COVID-19 has become a long-term event that is distinct from such disasters as hurricanes or terrorism events and more like the opioid crisis itself. Future research could examine COVID-19 literature to understand how health system approaches change over the course of such longer term disasters. Readers may judge for themselves the degree to which strained healthcare settings and pandemic-focused public health responses emphasizing physical distancing, which do not adequately support PWOUD, remain salient in light of successive COVID-19 “waves” and emergence of new coronavirus variants of concern.

## Conclusions

This scoping review and NGT study uncovers how disasters impact PWOUD and offers suggestions for better serving PWOUD. Our contextualization of findings to Alberta may be useful as a guide for those considering contextualization of literature evidence to their own contexts. Informed approaches to addressing social determinants of health and patient needs are required for greater accountability to PWOUD early during emergent disasters. As a component of disaster preparedness, healthcare systems need to engage in planning for key patient populations such as PWOUD to ensure their care can be continued concurrent with the response to the disaster. Stakeholder contextualization of the literature to Alberta highlights gaps in multi-risk management, data and decision-making, and public health organizing to respond to heightened adversities for PWOUD early during the pandemic. Simultaneous attention to multiple crises, with adequate resources to allow attention for both social and health systems issues, can prepare a system to serve PWOUD during disasters.

## Supplementary Information

**Additional file 1.** Supplementary Methods; this document provides the full search strategy including search terms

**Additional file 2.** Supplementary Results of Scientific Literature; this document provides a full list of the included scientific literature and brief overview of sources in a table with descriptions of pertinent findings

**Additional file 3.** Supplementary Results of Grey Literature, this document provides a brief summary of the grey literature, highlighting critical documents

**Additional file 4.** Literature Summary Sheet, this document provides infographics synthesizing the academic and grey literature according to three thematic areas (increased risk, risk mitigation, and cross-systems issues) that guided the NGT discussions

## Data Availability

All data generated or analysed during this study are included in this published article and its supplementary information files (methods, results 1 & 2 and literature summary sheets). No other datasets were generated during this study.

## Abbreviations

NGT: Nominal Group Technique
OAT: Opioid Agonist Therapy
OUD: Opioid Use Disorder
PPE: Personal Protective Equipment
PWOUD: People with Opioid Use Disorder
SCS: Supervised Consumption Site
