# Supplementary material for: Opioid use disorder treatment disruptions during the early COVID-19 pandemic and other emergent disasters: a scoping review addressing dual public health emergencies
Source: BMC Public Health. 2021 Jul 28;21:1471. doi: 10.1186/s12889-021-11495-0 (PMC8318046; doi:10.1186/s12889-021-11495-0)
Supplement: Supplementary file 1 — Additional file 1. Supplementary Methods; this document provides the full search strategy including search terms [file 12889_2021_11495_MOESM1_ESM.docx]

**Additional File 1: Supplementary Methods**

**TITLE:** Opioid Use Disorder Treatment Disruptions during the COVID-19 Pandemic and Other Disasters: A Scoping Review Addressing Dual Public Health Emergencies

**JOURNAL:** BMC Public Health

**AUTHOR NAMES:** Rita Henderson, Ashley McInnes, Leslee Mackey, Myles Bruised Head, Lindsay Crowshoe, Jessica Hann, Jake Hayward, Brian R. Holroyd, Eddy Lang, Bonnie Larson, Ashley Jane Leonard, Steven Persaud, Khalil Raghavji, Chris Sarin, Hakique Virani, Iskotoahka (William) Wadsworth, Stacey Whitman, Patrick McLane

**CORRESPONDING AUTHOR:** Rita Henderson, Department of Family Medicine, University of Calgary

Email: rihender@ucalgary.ca

**Peer-Reviewed Database Search Terms and Results**

| **#** | **Ovid MEDLINE(R) and In-Process & Other Non-Indexed Citations and Daily 1946 to June 03, 2020** | **Results** |
| --- | --- | --- |
| 1 | exp Pandemics/ or pandemic*.mp. | 29819 |
| 2 | exp Disease Outbreaks/ or "disease outbreak*".mp. | 101768 |
| 3 | exp Disasters/ or exp Natural Disasters/ or disaster*.mp. | 95436 |
| 4 | exp Coronavirus/ or exp Coronavirus Infections/ or exp Betacoronavirus/ or coronaviru*.mp. or "corona virus*".mp. or ncov*.mp. or n-cov*.mp. or COVID-19.mp. or COVID19.mp. or COVID-2019.mp. or COVID2019.mp. or SARS-COV-2.mp. or SARSCOV-2.mp. or SARSCOV2.mp. or SARSCOV19.mp. or Sars-Cov-19.mp. or SarsCov-19.mp. or SARSCOV2019.mp. or Sars-Cov-2019.mp. or SarsCov-2019.mp. or "severe acute respiratory syndrome cov 2".mp. or "2019 ncov".mp. or "2019ncov".mp. | 25637 |
| 5 | exp Influenza A Virus, H1N1 Subtype/ | 15158 |
| 6 | exp SARS Virus/ or SARS.mp. or exp Severe Acute Respiratory Syndrome/ or "severe acute respiratory syndrome".mp. or coronavirus.mp. [mp=title, abstract, original title, name of substance word, subject heading word, floating sub-heading word, keyword heading word, organism supplementary concept word, protocol supplementary concept word, rare disease supplementary concept word, unique identifier, synonyms] | 24675 |
| 7 | exp Middle East Respiratory Syndrome Coronavirus/ or ("Middle East Respiratory Syndrome Coronavirus" or "MERS virus" or "MERS Viruses" or "MERS-CoV" or "Novel Coronavirus").mp. | 3385 |
| 8 | 1 or 2 or 3 or 4 or 5 or 6 or 7 | 237927 |
| 9 | exp Analgesics, Opioid/ or exp Opioid-Related Disorders/ or "opioid use".mp. or "opioid usage".mp. or opioid*.mp. [mp=title, abstract, original title, name of substance word, subject heading word, floating sub-heading word, keyword heading word, organism supplementary concept word, protocol supplementary concept word, rare disease supplementary concept word, unique identifier, synonyms] | 175566 |
| 10 | *Substance-Related Disorders/ | 69015 |
| 11 | ("opioid user*" or "opioid addict*").mp. [mp=title, abstract, original title, name of substance word, subject heading word, floating sub-heading word, keyword heading word, organism supplementary concept word, protocol supplementary concept word, rare disease supplementary concept word, unique identifier, synonyms] | 2511 |
| 12 | Drug Users/ or "drug user*".mp. [mp=title, abstract, original title, name of substance word, subject heading word, floating sub-heading word, keyword heading word, organism supplementary concept word, protocol supplementary concept word, rare disease supplementary concept word, unique identifier, synonyms] | 18112 |
| 13 | ("substance use disorder*" or SUD).mp. [mp=title, abstract, original title, name of substance word, subject heading word, floating sub-heading word, keyword heading word, organism supplementary concept word, protocol supplementary concept word, rare disease supplementary concept word, unique identifier, synonyms] | 15049 |
| 14 | narcotic*.mp. or exp Narcotics/ | 155888 |
| 15 | 9 or 10 or 11 or 12 or 13 or 14 | 284584 |
| 16 | exp "Delivery of Health Care"/ | 1069135 |
| 17 | exp Emergency Service, Hospital/ or "emergency service*".mp. [mp=title, abstract, original title, name of substance word, subject heading word, floating sub-heading word, keyword heading word, organism supplementary concept word, protocol supplementary concept word, rare disease supplementary concept word, unique identifier, synonyms] | 83960 |
| 18 | exp Emergency Responders/ or exp Emergency Medical Technicians/ or exp Emergency Medical Services/ | 147791 |
| 19 | ("urgent care" or "urgent care cent*").mp. [mp=title, abstract, original title, name of substance word, subject heading word, floating sub-heading word, keyword heading word, organism supplementary concept word, protocol supplementary concept word, rare disease supplementary concept word, unique identifier, synonyms] | 2019 |
| 20 | exp Inpatients/ or inpatient*.mp. | 113115 |
| 21 | exp Hospitalization/ or hospitalisation.mp. [mp=title, abstract, original title, name of substance word, subject heading word, floating sub-heading word, keyword heading word, organism supplementary concept word, protocol supplementary concept word, rare disease supplementary concept word, unique identifier, synonyms] | 246936 |
| 22 | community health centers/ or substance abuse treatment centers/ or community mental health centers/ | 15182 |
| 23 | ("community health centre*" or "substance abuse treatment centre*" or "community mental health centre*").mp. [mp=title, abstract, original title, name of substance word, subject heading word, floating sub-heading word, keyword heading word, organism supplementary concept word, protocol supplementary concept word, rare disease supplementary concept word, unique identifier, synonyms] | 1147 |
| 24 | ("private addiction care" or "public addiction care").mp. [mp=title, abstract, original title, name of substance word, subject heading word, floating sub-heading word, keyword heading word, organism supplementary concept word, protocol supplementary concept word, rare disease supplementary concept word, unique identifier, synonyms] | 1 |
| 25 | exp Primary Health Care/ | 157690 |
| 26 | ("general practitioner*" or GP).mp. [mp=title, abstract, original title, name of substance word, subject heading word, floating sub-heading word, keyword heading word, organism supplementary concept word, protocol supplementary concept word, rare disease supplementary concept word, unique identifier, synonyms] | 83514 |
| 27 | exp Physicians, Family/ | 16336 |
| 28 | 16 or 17 or 18 or 19 or 20 or 21 or 22 or 23 or 24 or 25 or 26 or 27 | 1572314 |
| 29 | (distrupt* or chang* or cancel* or impact* or outcome* or "lack of healthcare").mp. [mp=title, abstract, original title, name of substance word, subject heading word, floating sub-heading word, keyword heading word, organism supplementary concept word, protocol supplementary concept word, rare disease supplementary concept word, unique identifier, synonyms] | 5840076 |
| 30 | exp Health Facility Closure/ or closure*.mp. [mp=title, abstract, original title, name of substance word, subject heading word, floating sub-heading word, keyword heading word, organism supplementary concept word, protocol supplementary concept word, rare disease supplementary concept word, unique identifier, synonyms] | 108168 |
| 31 | exp Health Services Accessibility/ or access*.mp. | 582378 |
| 32 | exp Telemedicine/ or telecare.mp. | 28177 |
| 33 | exp Infection Control/ or "infection control and prevention".mp. | 63485 |
| 34 | exp Internet/ or exp Digital Divide/ or "digital divide".mp. or "internet access".mp. [mp=title, abstract, original title, name of substance word, subject heading word, floating sub-heading word, keyword heading word, organism supplementary concept word, protocol supplementary concept word, rare disease supplementary concept word, unique identifier, synonyms] | 79471 |
| 35 | ("virtual system*" or "virtual process*" or "virtual healthcare").mp. [mp=title, abstract, original title, name of substance word, subject heading word, floating sub-heading word, keyword heading word, organism supplementary concept word, protocol supplementary concept word, rare disease supplementary concept word, unique identifier, synonyms] | 159 |
| 36 | exp Fear/ or "patient fear*".mp. | 32796 |
| 37 | ("community clinic" adj2 closure).mp. [mp=title, abstract, original title, name of substance word, subject heading word, floating sub-heading word, keyword heading word, organism supplementary concept word, protocol supplementary concept word, rare disease supplementary concept word, unique identifier, synonyms] | 0 |
| 38 | ("addiction clinic*" adj2 closure).mp. [mp=title, abstract, original title, name of substance word, subject heading word, floating sub-heading word, keyword heading word, organism supplementary concept word, protocol supplementary concept word, rare disease supplementary concept word, unique identifier, synonyms] | 0 |
| 39 | ("lack of healthcare" or "healthcare restriction*").mp. [mp=title, abstract, original title, name of substance word, subject heading word, floating sub-heading word, keyword heading word, organism supplementary concept word, protocol supplementary concept word, rare disease supplementary concept word, unique identifier, synonyms] | 119 |
| 40 | ("social distanc*" or "physical distanc*").mp. [mp=title, abstract, original title, name of substance word, subject heading word, floating sub-heading word, keyword heading word, organism supplementary concept word, protocol supplementary concept word, rare disease supplementary concept word, unique identifier, synonyms] | 4900 |
| 41 | exp Social Behavior/ or social behaviour*.mp. | 253611 |
| 42 | "social risk factor*".mp. [mp=title, abstract, original title, name of substance word, subject heading word, floating sub-heading word, keyword heading word, organism supplementary concept word, protocol supplementary concept word, rare disease supplementary concept word, unique identifier, synonyms] | 650 |
| 43 | exp Quarantine/ or quarantine.mp. | 5467 |
| 44 | exp Social Isolation/ or "social isolation".mp. or isolation.mp. | 1163137 |
| 45 | exp Loneliness/ or loneliness.mp. | 6845 |
| 46 | 29 or 30 or 31 or 32 or 33 or 34 or 35 or 36 or 37 or 38 or 39 or 40 or 41 or 42 or 43 or 44 or 45 | 7617518 |
| 47 | 8 and 15 and 28 and 46 | 200 |
| 48 | limit 47 to yr="2000 -Current" | 169 |

| CINAHL Complete  Searched: June 2 2020 | | | # of results |
| --- | --- | --- | --- |
| S1 | (MH "Disease Outbreaks") OR "pandemic* or epidemic* or outbreak*" | Expanders - Apply equivalent subjects  Search modes - Boolean/Phrase | 29,836 |
| S2 | (MH "Natural Disasters") OR (MH "Disasters+") OR "disaster*" | Expanders - Apply equivalent subjects  Search modes - Boolean/Phrase | 39,521 |
| S3 | (MH "Coronavirus Infections+") OR (MH "Coronavirus+") | Expanders - Apply equivalent subjects  Search modes - Boolean/Phrase | 3,874 |
| S4 | Betacoronavirus OR coronaviru* OR "corona virus*" OR ncov* OR n-cov* OR COVID-19 OR COVID19 OR "COVID-2019" OR COVID2019 OR "SARS-COV-2" OR "SARSCOV-2" OR SARSCOV2 OR SARSCOV19 OR "Sars-Cov-19" OR "SarsCov-19" OR SARSCOV2019 OR "Sars-Cov-2019" OR "SarsCov-2019" OR "severe acute respiratory syndrome cov 2"OR "2019 ncov" OR "2019ncov" | Expanders - Apply equivalent subjects  Search modes - Boolean/Phrase | 5,446 |
| S5 | (MH "Influenza A Virus, H1N1 Subtype") | Expanders - Apply equivalent subjects  Search modes - Boolean/Phrase | 3,410 |
| S6 | (MH "SARS Virus") OR (MH "Severe Acute Respiratory Syndrome") OR "severe acute respiratory syndrome" | Expanders - Apply equivalent subjects  Search modes - Boolean/Phrase | 2,703 |
| S7 | (MH "Middle East Respiratory Syndrome Coronavirus") OR (MH "Middle East Respiratory Syndrome") | Expanders - Apply equivalent subjects  Search modes - Boolean/Phrase | 597 |
| S8 | "Middle East Respiratory Syndrome Coronavirus" OR "MERS virus" OR "MERS Viruses" OR "MERS-CoV" OR "Novel Coronavirus" | Expanders - Apply equivalent subjects  Search modes - Boolean/Phrase | 1,075 |
| S9 | S1 OR S2 OR S3 OR S4 OR S5 OR S6 OR S7 OR S8 | Expanders - Apply equivalent subjects  Search modes - Boolean/Phrase | 73,675 |
| S10 | (MH "Narcotics+") OR (MH "Analgesics, Opioid+") OR "opioid*" | Expanders - Apply equivalent subjects  Search modes - Boolean/Phrase | 64,917 |
| S11 | (MM "Substance Use Disorders") | Expanders - Apply equivalent subjects  Search modes - Boolean/Phrase | 28,836 |
| S12 | "opioid use" OR "opioid usage" | Expanders - Apply equivalent subjects  Search modes - Boolean/Phrase | 5,710 |
| S13 | "opioid user*" OR "opioid addict*" | Expanders - Apply equivalent subjects  Search modes - Boolean/Phrase | 1,601 |
| S14 | (MH "Substance Abusers+") OR ""drug user*"" OR (MH "Street Drugs+") | Expanders - Apply equivalent subjects  Search modes - Boolean/Phrase | 37,341 |
| S15 | "substance use disorder*" OR SUD | Expanders - Apply equivalent subjects  Search modes - Boolean/Phrase | 42,070 |
| S16 | ""opioid use disorder*"" | Expanders - Apply equivalent subjects  Search modes - Boolean/Phrase | 2,124 |
| S17 | S10 OR S11 OR S12 OR S13 OR S14 OR S15 OR S16 | Expanders - Apply equivalent subjects  Search modes - Boolean/Phrase | 124,223 |
| S18 | (MH "Health Care Delivery+") | Expanders - Apply equivalent subjects  Search modes - Boolean/Phrase | 355,169 |
| S19 | (MH "Emergency Service+") OR (MH "Emergency Medical Services+") OR "emergency service*" | Expanders - Apply equivalent subjects  Search modes - Boolean/Phrase | 108,929 |
| S20 | "Emergency Responder*" | Expanders - Apply equivalent subjects  Search modes - Boolean/Phrase | 298 |
| S21 | paramedic* or ems or prehospital or pre-hospital or ambulance or emergency medical technician* or emt | Expanders - Apply equivalent subjects  Search modes - Boolean/Phrase | 42,431 |
| S22 | "accident or emergency" OR A&E | Expanders - Apply equivalent subjects  Search modes - Boolean/Phrase | 109,867 |
| S23 | "urgent care" OR "urgent care cent*" | Expanders - Apply equivalent subjects  Search modes - Boolean/Phrase | 8,600 |
| S24 | (MH "Inpatients") OR "inpatient or acute or hospital or ward or unit" | Expanders - Apply equivalent subjects  Search modes - Boolean/Phrase | 89,383 |
| S25 | (MH "Hospitalization+")OR hospitalisation | Expanders - Apply equivalent subjects  Search modes - Boolean/Phrase | 145,041 |
| S26 | (MH "Community Health Centers+") OR (MH "Rural Health Centers") OR (MH "Community Mental Health Services+") OR (MH "Hospitals, Community") | Expanders - Apply equivalent subjects  Search modes - Boolean/Phrase | 25,365 |
| S27 | "community health centre*" | Expanders - Apply equivalent subjects  Search modes - Boolean/Phrase | 2,981 |
| S28 | (MH "Substance Use Rehabilitation Programs+") OR "substance abuse treatment cent*" | Expanders - Apply equivalent subjects  Search modes - Boolean/Phrase | 14,847 |
| S29 | ""private addiction care" OR "public addiction care"" | Expanders - Apply equivalent subjects  Search modes - Boolean/Phrase | 5,583 |
| S30 | ""private addiction care" OR "public addiction care"" | Expanders - Apply equivalent subjects  Search modes - SmartText Searching | 5,583 |
| S31 | (MH "Primary Health Care") | Expanders - Apply equivalent subjects  Search modes - Boolean/Phrase | 66,685 |
| S32 | "general practitioner*" or gp | Expanders - Apply equivalent subjects  Search modes - Boolean/Phrase | 35,544 |
| S33 | (MH "Physicians, Family") | Expanders - Apply equivalent subjects  Search modes - Boolean/Phrase | 20,520 |
| S34 | S18 OR S19 OR S20 OR S21 OR S22 OR S23 OR S24 OR S25 OR S26 OR S27 OR S28 OR S29 OR S30 OR S31 OR S32 OR S33 | Expanders - Apply equivalent subjects  Search modes - Boolean/Phrase | 830,205 |
| S35 | distrupt* OR chang* OR cancel* OR impact* OR outcome* OR "lack of healthcare" | Expanders - Apply equivalent subjects  Search modes - Boolean/Phrase | 1,682,794 |
| S36 | (MH "Health Facility Closure+") OR Closure* | Expanders - Apply equivalent subjects  Search modes - Boolean/Phrase | 22,422 |
| S37 | (MH "Health Services Accessibility+") OR access* | Expanders - Apply equivalent subjects  Search modes - Boolean/Phrase | 254,665 |
| S38 | (MH "Telemedicine+") OR "telecare or telehealth or telemedicine" OR (MH "Telehealth+") | Expanders - Apply equivalent subjects  Search modes - Boolean/Phrase | 24,143 |
| S39 | (MH "Infection Control+") OR "Infection prevention Control" | Expanders - Apply equivalent subjects  Search modes - Boolean/Phrase | 76,586 |
| S40 | (MH "Digital Divide") OR ""digital divide"" | Expanders - Apply equivalent subjects  Search modes - Boolean/Phrase | 747 |
| S41 | (MH "Internet Access") OR ""internet access"" | Expanders - Apply equivalent subjects  Search modes - Boolean/Phrase | 1,038 |
| S42 | "virtual system*" OR "virtual process*" OR "virtual healthcare" | Expanders - Apply equivalent subjects  Search modes - Boolean/Phrase | 54 |
| S43 | ""patient fear*"" | Expanders - Apply equivalent subjects  Search modes - Boolean/Phrase | 463 |
| S44 | (MH "Fear+") | Expanders - Apply equivalent subjects  Search modes - Boolean/Phrase | 15,163 |
| S45 | "community clinic" N3 closure | Expanders - Apply equivalent subjects  Search modes - Boolean/Phrase | 2,708 |
| S46 | "community clinic" N3 closure | Expanders - Apply equivalent subjects  Search modes - SmartText Searching | 2,708 |
| S47 | "addiction clinic*" N2 closure | Expanders - Apply equivalent subjects  Search modes - Boolean/Phrase | 2,052 |
| S48 | "addiction clinic*" N2 closure | Expanders - Apply equivalent subjects  Search modes - SmartText Searching | 2,052 |
| S49 | "lack of healthcare" or "healthcare restriction*" | Expanders - Apply equivalent subjects  Search modes - Boolean/Phrase | 64 |
| S50 | "social distanc*" or "physical distanc*" | Expanders - Apply equivalent subjects  Search modes - Boolean/Phrase | 904 |
| S51 | (MH "Social Behavior+") OR "social behaviour*" | Expanders - Apply equivalent subjects  Search modes - Boolean/Phrase | 45,385 |
| S52 | ""social risk factor*"" | Expanders - Apply equivalent subjects  Search modes - Boolean/Phrase | 336 |
| S53 | (MH "Quarantine") OR quarantine | Expanders - Apply equivalent subjects  Search modes - Boolean/Phrase | 939 |
| S54 | (MH "Social Isolation+") OR "social isolation" | Expanders - Apply equivalent subjects  Search modes - Boolean/Phrase | 15,217 |
| S55 | "isolation" | Expanders - Apply equivalent subjects  Search modes - Boolean/Phrase | 32,389 |
| S56 | "loneliness" | Expanders - Apply equivalent subjects  Search modes - Boolean/Phrase | 6,579 |
| S57 | (MH "Loneliness") | Expanders - Apply equivalent subjects  Search modes - Boolean/Phrase | 4,566 |
| S58 | S35 OR S36 OR S37 OR S38 OR S39 OR S40 OR S41 OR S42 OR S43 OR S44 OR S45 OR S46 OR S47 OR S48 OR S49 OR S50 OR S51 OR S52 OR S53 OR S54 OR S55 OR S56 OR S57 | Expanders - Apply equivalent subjects  Search modes - Boolean/Phrase | 2,012,845 |
| S59 | S9 AND S17 AND S34 AND S58 | Expanders - Apply equivalent subjects  Search modes - Boolean/Phrase | 107 |
| S60 | S9 AND S17 AND S34 AND S58 | Limiters - Published Date: 20000101-20201231  Expanders - Apply equivalent subjects  Search modes - Boolean/Phrase | 106 |

| **#** | **APA PsycInfo 1806 to May Week 4 2020** | **Results** |
| --- | --- | --- |
| 1 | exp Pandemics/ or pandemic*.mp. | 1956 |
| 2 | exp Disease Outbreaks/ or "disease outbreak*".mp. | 4223 |
| 3 | exp Disasters/ or exp Natural Disasters/ or disaster*.mp. | 14601 |
| 4 | exp Coronavirus/ or exp Coronavirus Infections/ or exp Betacoronavirus/ or coronaviru*.mp. or "corona virus*".mp. or ncov*.mp. or n-cov*.mp. or COVID-19.mp. or COVID19.mp. or COVID-2019.mp. or COVID2019.mp. or SARS-COV-2.mp. or SARSCOV-2.mp. or SARSCOV2.mp. or SARSCOV19.mp. or Sars-Cov-19.mp. or SarsCov-19.mp. or SARSCOV2019.mp. or Sars-Cov-2019.mp. or SarsCov-2019.mp. or "severe acute respiratory syndrome cov 2".mp. or "2019 ncov".mp. or "2019ncov".mp. | 239 |
| 5 | exp Swine Influenza/ or H1N1.mp. | 524 |
| 6 | exp Middle East Respiratory Syndrome Coronavirus/ or ("Middle East Respiratory Syndrome Coronavirus" or "MERS virus" or "MERS Viruses" or "MERS-CoV" or "Novel Coronavirus").mp. | 35 |
| 7 | 1 or 2 or 3 or 4 or 5 or 6 | 20191 |
| 8 | exp Opiates/ or exp Drug Abuse/ or exp "Opioid Use Disorder"/ or opioid*.mp. or exp "Substance Use Disorder"/ | 152083 |
| 9 | ("opioid use" or "opioid usage*").mp. [mp=title, abstract, heading word, table of contents, key concepts, original title, tests & measures, mesh] | 3204 |
| 10 | ("substance use disorder" or SUD).mp. [mp=title, abstract, heading word, table of contents, key concepts, original title, tests & measures, mesh] | 11211 |
| 11 | ("opioid user*" or "opioid addict*").mp. [mp=title, abstract, heading word, table of contents, key concepts, original title, tests & measures, mesh] | 1443 |
| 12 | exp Drug Abuse/ or exp Drug Addiction/ or exp Drug Usage/ or "drug user*".mp. | 154200 |
| 13 | narcotic*.mp. or exp Narcotic Drugs/ | 34986 |
| 14 | 8 or 9 or 10 or 11 or 12 or 13 | 227499 |
| 15 | exp Health Care Delivery/ | 97373 |
| 16 | exp Emergency Services/ or "emergency service*".mp. | 13008 |
| 17 | Emergency Responder*.mp. or exp First Responders/ | 471 |
| 18 | exp Paramedical Sciences/ or exp Paramedics/ or Emergency Medical Technicians.mp. | 50897 |
| 19 | ("urgent care" or "urgent care cent*").mp. [mp=title, abstract, heading word, table of contents, key concepts, original title, tests & measures, mesh] | 305 |
| 20 | exp Hospitalized Patients/ or exp Psychiatric Hospitalization/ or inpatient*.mp. | 64436 |
| 21 | exp Community Mental Health Services/ or exp Community Health/ or exp Community Mental Health Centers/ or exp Community Facilities/ or community health center*.mp. | 26692 |
| 22 | exp "Substance Use Treatment"/ or substance abuse treatment centers.mp. | 30444 |
| 23 | ("community mental health centre*" or "substance abuse treatment centre*" or "community health centre*").mp. [mp=title, abstract, heading word, table of contents, key concepts, original title, tests & measures, mesh] | 350 |
| 24 | exp Addiction Treatment/ | 33787 |
| 25 | ("private addiction care" or "public addiction care").mp. [mp=title, abstract, heading word, table of contents, key concepts, original title, tests & measures, mesh] | 1 |
| 26 | exp Primary Health Care/ | 18137 |
| 27 | exp General Practitioners/ or GP.mp. | 8573 |
| 28 | "family physician*".mp. or exp Family Physicians/ | 3088 |
| 29 | 15 or 16 or 17 or 18 or 19 or 20 or 21 or 22 or 23 or 24 or 25 or 26 or 27 or 28 | 284686 |
| 30 | (distrupt* or chang* or cancel* or impact* or outcome* or "lack of healthcare").mp. [mp=title, abstract, heading word, table of contents, key concepts, original title, tests & measures, mesh] | 1264410 |
| 31 | Closure*.mp. | 7391 |
| 32 | "Health Facility Closure".mp. | 150 |
| 33 | exp Health Care Access/ or Health Services Accessibility.mp. or access*.mp. | 154332 |
| 34 | exp Telemedicine/ | 8629 |
| 35 | Telehealth.mp. or exp Telemedicine/ | 8958 |
| 36 | "Infection prevention Control".mp. | 6 |
| 37 | Infection Control.mp. | 564 |
| 38 | infection prevention.mp. | 269 |
| 39 | "digital divide".mp. or exp Digital Divide/ | 1142 |
| 40 | exp Internet Usage/ or exp Internet/ or Internet Access.mp. | 39465 |
| 41 | ("virtual system*" or "virtual process*" or "virtual healthcare").mp. [mp=title, abstract, heading word, table of contents, key concepts, original title, tests & measures, mesh] | 52 |
| 42 | exp Fear/ or "patient fear*".mp. | 20426 |
| 43 | ("community clinic" adj2 closure).mp. [mp=title, abstract, heading word, table of contents, key concepts, original title, tests & measures, mesh] | 0 |
| 44 | ("addiction clinic*" adj2 closure).mp. [mp=title, abstract, heading word, table of contents, key concepts, original title, tests & measures, mesh] | 0 |
| 45 | ("lack of healthcare" or "healthcare restriction*").mp. [mp=title, abstract, heading word, table of contents, key concepts, original title, tests & measures, mesh] | 33 |
| 46 | ("social distanc*" or "physical distanc*").mp. [mp=title, abstract, heading word, table of contents, key concepts, original title, tests & measures, mesh] | 4632 |
| 47 | exp Social Behavior/ or "social behaviour*".mp. | 1101180 |
| 48 | "social risk factor*".mp. | 456 |
| 49 | quarantine.mp. | 220 |
| 50 | "social isolation".mp. or exp Social Isolation/ | 15718 |
| 51 | isolation.mp. | 32197 |
| 52 | loneliness.mp. or exp Loneliness/ | 11161 |
| 53 | 30 or 31 or 32 or 33 or 34 or 35 or 36 or 37 or 38 or 39 or 40 or 41 or 42 or 43 or 44 or 45 or 46 or 47 or 48 or 49 or 50 or 51 or 52 | 2172464 |
| 54 | 7 and 14 and 29 and 53 | 102 |
| 55 | limit 54 to yr="2000 -Current" | 97 |

| **Science Direct / Science Direct Covid -19 Research/ Elsevier 1Science Coronavirus Research Repository**  Searched: June 4 2020 | | **# of results** |
| --- | --- | --- |
| 1 | Database Covid-19 & other pandemic filter was used to search: "COVID-19" OR Coronavirus OR "Corona virus" OR Coronaviruses OR "2019-nCoV" OR "SARS-CoV" OR "MERS-CoV" OR “Severe Acute Respiratory Syndrome” OR “Middle East Respiratory AND "opioid addiction" OR "opioid-use disorder" OR opioid OR "substance-use disorder" OR "substance-use" | 136 results |
|  | "natural disaster" OR disaster OR "disease outbreak" OR pandemic AND "opioid addiction" OR "opioid-use disorder" OR opioids OR opioid [limited to 2000-2020] | 118 |
|  | "natural disaster" OR disaster OR "disease outbreak" OR pandemic OR "substance-use disorder" [limited to 2000-2020] | 115 |
| **Elsevier 1Science Coronavirus Research Repository** | | |
| 2 | ("opioid addiction" OR "opioid-use disorder" OR opioid OR "substance-use disorder" OR "substance-use") | 60 |

| **LitCovid Database**  Searched 2 June 2020 | | **# of results** |
| --- | --- | --- |
| LitCovid *the search strategy had to be amended for this database. The two example searches below show the large amount of results returned when multiple concepts are used. It is for this reason that a broad search only focusing on the opioid concept was carried out, with the first 100 results being included (the relevant-looking results started to disappear around page 4 onwards)  Covid-19 filter already exists over-top of this database so it is not needed to be searched as a concept | | |
| 1 | emergency medical services or emergency medicine or emergency medical service communication system or ems or prehospital or pre-hospital or “out of hospital” or emergency medical technicians or ems or emt or paramedic or paramedics or paramedicine or community health workers or emergency technician or advance care provider or advanced care provider or emergency dispatch or dispatch or emergency practitioner or ambulances or air ambulances or ambulance or aeromedic or ambulatory care or emergency treatment or critical care or subacute care or acute care AND opioids OR opioid OR "opioid-related disorders" OR substance-related disorders" OR “opioid use” OR “opioid users” OR “opioid addiction” OR “substance use disorder" OR SUD OR "drug user” OR “drug users” OR narcotic OR narcotics AND disruption OR challenge OR outcome OR change OR “access to healthcare” OR “health facility closure” OR closures OR access OR cancellation OR “patient fear” OR fear OR “infection prevention” OR internet OR “internet access” OR “digital divide” OR telehealth OR telemedicine OR “social isolation” OR isolation OR “physical distancing” OR “social distancing” OR loneliness OR “heath care restriction” OR “social behaviour” OR “social behavior” OR quarantine | 14397 |
|  | disrupt* OR chang* OR cancel* OR impact* OR outcome* OR challenge* AND opioids OR opioid OR "opioid-related disorders" OR substance-related disorders" OR “opioid use” OR “opioid users” OR “opioid addiction” OR “substance use disorder" OR SUD OR "drug user” OR “drug users” OR narcotic OR narcotics | 6601 |
| 1 | (opioids OR opioid OR "opioid-related disorders" OR substance-related disorders" OR “opioid use” OR “opioid users” OR “opioid addiction” OR “substance use disorder" OR SUD) | First 100 results included  1849 |
| 2 | narcotic OR narcotics | 1 result |

| **WHO: Global Research on COVID-19**  As there was already a COVID-19 filter in use, I decided to do a more focused search on opioids only as well as those including medical environments. As you can see, including the other search concepts doesn’t bring back any results  Searched June 4 2020 | | # of results |
| --- | --- | --- |
| 1 | opioids OR opioid OR "opioid-related disorders" OR substance-related disorders" OR “opioid use” OR “opioid users” OR “opioid addiction” OR “substance use disorder" OR SUD OR "drug user” OR “drug users” OR narcotic OR narcotics | 165 |
| 2 | opioids OR opioid OR "opioid-related disorders" OR substance-related disorders" OR “opioid use” OR “opioid users” OR “opioid addiction” OR “substance use disorder" OR SUD OR "drug user” OR “drug users” OR narcotic OR narcotics AND "emergency medical services" OR EMS OR "first responders" OR "emergency medical technician" AND disruption OR disruptions OR changes OR cancellations OR cancelation OR impact OR challenge OR challenges OR outcome impact* or "lack of healthcare" OR closure OR closures OR access OR telemedicine OR "digital divide" OR internet OR fear OR "patient fear" OR "social distancing" OR "physical distancing" OR "social risk factor" OR "social behaviour" OR "social behavior" OR isolation OR "social isolation" OR quarantine OR loneliness | 0 |
| 3 | opioids OR opioid OR "opioid-related disorders" OR substance-related disorders" OR “opioid use” OR “opioid users” OR “opioid addiction” OR “substance use disorder" OR SUD OR "drug user” OR “drug users” OR narcotic OR narcotics AND Inpatient OR inpatients AND disruption OR disruptions OR changes OR cancellations OR cancelation OR impact OR challenge OR challenges OR outcome impact* or "lack of healthcare" OR closure OR closures OR access OR telemedicine OR "digital divide" OR internet OR fear OR "patient fear" OR "social distancing" OR "physical distancing" OR "social risk factor" OR "social behaviour" OR "social behavior" OR isolation OR "social isolation" OR quarantine OR loneliness | 0 |
| 4 | opioids OR opioid OR "opioid-related disorders" OR substance-related disorders" OR “opioid use” OR “opioid users” OR “opioid addiction” OR “substance use disorder" OR SUD OR "drug user” OR “drug users” OR narcotic OR narcotics AND hospitalisation OR hospitalization AND disruption OR disruptions OR changes OR cancellations OR cancelation OR impact OR challenge OR challenges OR outcome impact* or "lack of healthcare" OR closure OR closures OR access OR telemedicine OR "digital divide" OR internet OR fear OR "patient fear" OR "social distancing" OR "physical distancing" OR "social risk factor" OR "social behaviour" OR "social behavior" OR isolation OR "social isolation" OR quarantine OR loneliness | 0 |
| 5 | opioids OR opioid OR "opioid-related disorders" OR substance-related disorders" OR “opioid use” OR “opioid users” OR “opioid addiction” OR “substance use disorder" OR SUD OR "drug user” OR “drug users” OR narcotic OR narcotics AND “urgent care” AND disruption OR disruptions OR changes OR cancellations OR cancelation OR impact OR challenge OR challenges OR outcome impact* or "lack of healthcare" OR closure OR closures OR access OR telemedicine OR "digital divide" OR internet OR fear OR "patient fear" OR "social distancing" OR "physical distancing" OR "social risk factor" OR "social behaviour" OR "social behavior" OR isolation OR "social isolation" OR quarantine OR loneliness | 0 |
| 6 | opioids OR opioid OR "opioid-related disorders" OR substance-related disorders" OR “opioid use” OR “opioid users” OR “opioid addiction” OR “substance use disorder" OR SUD OR "drug user” OR “drug users” OR narcotic OR narcotics AND “community Health Center” OR “community health centers” OR “community health centre” OR “community health centres” OR “community Mental Health Services” OR “community Mental Health Services” OR “Substance Use Rehabilitation Program” OR “Substance Use Rehabilitation Programs” OR “addiction treatment” OR “substance abuse treatment” AND disruption OR disruptions OR changes OR cancellations OR cancelation OR impact OR challenge OR challenges OR outcome impact* or "lack of healthcare" OR closure OR closures OR access OR telemedicine OR "digital divide" OR internet OR fear OR "patient fear" OR "social distancing" OR "physical distancing" OR "social risk factor" OR "social behaviour" OR "social behavior" OR isolation OR "social isolation" OR quarantine OR loneliness | 0 |
| 7 | opioids OR opioid OR "opioid-related disorders" OR substance-related disorders" OR “opioid use” OR “opioid users” OR “opioid addiction” OR “substance use disorder" OR SUD OR "drug user” OR “drug users” OR narcotic OR narcotics AND “primary health care” OR general practitioner” OR GP OR “family physician” AND opioids OR opioid OR "opioid-related disorders" OR substance-related disorders" OR “opioid use” OR “opioid users” OR “opioid addiction” OR “substance use disorder" OR SUD OR "drug user” OR “drug users” OR narcotic OR narcotics AND “community Health Center” OR “community health centers” OR “community health centre” OR “community health centres” OR “community Mental Health Services” OR “community Mental Health Services” OR “Substance Use Rehabilitation Program” OR “Substance Use Rehabilitation Programs” OR “addiction treatment” OR “substance abuse treatment” AND disruption OR disruptions OR changes OR cancellations OR cancelation OR impact OR challenge OR challenges OR outcome impact* or "lack of healthcare" OR closure OR closures OR access OR telemedicine OR "digital divide" OR internet OR fear OR "patient fear" OR "social distancing" OR "physical distancing" OR "social risk factor" OR "social behaviour" OR "social behavior" OR isolation OR "social isolation" OR quarantine OR loneliness | 0 |

| **TripPro**  Searched June 8 2020 | | # of results |
| --- | --- | --- |
|  | ("coronavirus" OR "coronavirus infections" OR "coronaviru*" OR "corona virus" OR "ncov*" OR "n cov*" OR "novel cov" OR "COVID-19" OR "COVID19" OR "COVID-2019" OR "COVID2019" OR "SARS-COV-2" OR "SARSCOV-2" OR "sarscov2" OR "SARSCOV19" OR "sars cov 19" OR "severe acute respiratory syndrome cov 2" OR "2019 ncov" OR "2019ncov" OR "severe acute respiratory disease") AND (opioid* OR "opioid-related disorder*" OR "substance-related disorder*" OR "opioid use" OR "opioid user*" OR "opioid addiction*" OR "substance use disorder*" OR SUD OR "drug user*" OR narcotic*) AND ("Emergency Service*" OR "Emergency Responder*" OR "First Responder*" OR "Paramedical Science*" OR Paramedic* OR "Emergency Medical Technician*" OR "urgent care" OR "Hospitalized Patient*" OR inpatient* OR "Community Mental Health Service* OR "Community Mental Health Center*" or "community health center*" OR "Substance Use Treatment*" OR "substance abuse treatment center*" OR "addiction treatment*" OR "community mental health centre*" OR "substance abuse treatment centre*" OR "community health centre*" OR "Primary Health Care" OR "General Practitioner*" OR "family physician") AND (disrupt* OR change* OR cancel* OR impact* OR challenge* OR outcome* OR "lack of healthcare" OR closure* OR access OR telemedicine OR "digital divide" OR internet OR fear OR "patient fear" OR "social distancing" OR "physical distancing" OR "social risk factor*" OR "social behaviour*" OR "social behavior*" OR isolation OR "social isolation" OR quarantine OR loneliness) | 39 |
|  | ("severe acute respiratory syndrome" OR "sars virus*" OR SARS OR "SARS-Cov") AND (opioid* OR "opioid-related disorder*" OR "substance-related disorder*" OR "opioid use" OR "opioid user*" OR "opioid addiction*" OR "substance use disorder*" OR SUD OR "drug user*" OR narcotic*) AND ("Emergency Service*" OR "Emergency Responder*" OR "First Responder*" OR "Paramedical Science*" OR Paramedic* OR "Emergency Medical Technician*" OR "urgent care" OR "Hospitalized Patient*" OR inpatient* OR "Community Mental Health Service*" OR "Community Mental Health Center*" or "community health center*" OR "Substance Use Treatment*" OR "substance abuse treatment center*" OR "addiction treatment*" OR "community mental health centre*" OR "substance abuse treatment centre*" OR "community health centre*" OR "Primary Health Care" OR "General Practitioner*" OR "family physician") AND (disrupt* OR change* OR cancel* OR impact* OR challenge* OR outcome* OR "lack of healthcare" OR closure* OR access OR telemedicine OR "digital divide" OR internet OR fear OR "patient fear" OR "social distancing" OR "physical distancing" OR "social risk factor*" OR "social behaviour*" OR "social behavior*" OR isolation OR "social isolation" OR quarantine OR loneliness) | 70 |
| 3 | ("middle east respiratory syndrome coronavirus" OR MERS OR "MERS-Cov") AND (opioid* OR "opioid-related disorder*" OR "substance-related disorder*" OR "opioid use" OR "opioid user*" OR "opioid addiction*" OR "substance use disorder*" OR SUD OR "drug user*" OR narcotic*) AND ("Emergency Service*" OR "Emergency Responder*" OR "First Responder*" OR "Paramedical Science*" OR Paramedic* OR "Emergency Medical Technician*" OR "urgent care" OR "Hospitalized Patient*" OR inpatient* OR "Community Mental Health Service*" OR "Community Mental Health Center*" or "community health center*" OR "Substance Use Treatment*" OR "substance abuse treatment center*" OR "addiction treatment*" OR "community mental health centre*" OR "substance abuse treatment centre*" OR "community health centre*" OR "Primary Health Care" OR "General Practitioner*" OR "family physician") AND (disrupt* OR change* OR cancel* OR impact* OR challenge* OR outcome* OR "lack of healthcare" OR closure* OR access OR telemedicine OR "digital divide" OR internet OR fear OR "patient fear" OR "social distancing" OR "physical distancing" OR "social risk factor*" OR "social behaviour*" OR "social behavior*" OR isolation OR "social isolation" OR quarantine OR loneliness) | 33 |
| 4 | ("influenza a virus, h1n1 subtype" OR H1N1 OR "swine flu") AND (opioid* OR "opioid-related disorder*" OR "substance-related disorder*" OR "opioid use" OR "opioid user*" OR "opioid addiction*" OR "substance use disorder*" OR SUD OR "drug user*" OR narcotic*) AND ("Emergency Service*" OR "Emergency Responder*" OR "First Responder*" OR "Paramedical Science*" OR Paramedic* OR "Emergency Medical Technician*" OR "urgent care" OR "Hospitalized Patient*" OR inpatient* OR "Community Mental Health Service*" OR "Community Mental Health Center*" or "community health center*" OR "Substance Use Treatment*" OR "substance abuse treatment center*" OR "addiction treatment*" OR "community mental health centre*" OR "substance abuse treatment centre*" OR "community health centre*" OR "Primary Health Care" OR "General Practitioner*" OR "family physician") AND (disrupt* OR change* OR cancel* OR impact* OR challenge* OR outcome* OR "lack of healthcare" OR closure* OR access OR telemedicine OR "digital divide" OR internet OR fear OR "patient fear" OR "social distancing" OR "physical distancing" OR "social risk factor*" OR "social behaviour*" OR "social behavior*" OR isolation OR "social isolation" OR quarantine OR loneliness) | 49 |
| 5 | ("pandemic" OR "disease outbreak*" OR disaster* OR "natural disaster*") AND (opioid* OR "opioid-related disorder*" OR "substance-related disorder*" OR "opioid use" OR "opioid user*" OR "opioid addiction*" OR "substance use disorder*" OR SUD OR "drug user*" OR narcotic*) AND ("Emergency Service*" OR "Emergency Responder*" OR "First Responder*" OR "Paramedical Science*" OR Paramedic* OR "Emergency Medical Technician*" OR "urgent care" OR "Hospitalized Patient*" OR inpatient* OR "Community Mental Health Service*" OR "Community Mental Health Center*" or "community health center*" OR "Substance Use Treatment*" OR "substance abuse treatment center*" OR "addiction treatment*" OR "community mental health centre*" OR "substance abuse treatment centre*" OR "community health centre*" OR "Primary Health Care" OR "General Practitioner*" OR "family physician") AND (disrupt* OR change* OR cancel* OR impact* OR challenge* OR outcome* OR "lack of healthcare" OR closure* OR access OR telemedicine OR "digital divide" OR internet OR fear OR "patient fear" OR "social distancing" OR "physical distancing" OR "social risk factor*" OR "social behaviour*" OR "social behavior*" OR isolation OR "social isolation" OR quarantine OR loneliness) | 259 |

| PubMed  Searched June 8 2020 | | # of results |
| --- | --- | --- |
| 1 | ((("ncov*"[Title/Abstract] OR "n cov*"[Title/Abstract] OR "novel cov"[Title/Abstract] OR "COVID-19"[Title/Abstract] OR "COVID19"[Title/Abstract] OR "COVID-2019"[Title/Abstract] OR "COVID2019"[Title/Abstract] OR "SARS-COV-2"[Title/Abstract] OR "SARSCOV-2"[Title/Abstract] OR "sarscov2 "[Title/Abstract] OR "SARSCOV19"[Title/Abstract] OR "sars cov 19 "[Title/Abstract] OR "severe acute respiratory syndrome cov 2"[Title/Abstract] OR "2019 ncov"[Title/Abstract] OR "2019ncov"[Title/Abstract] OR **"severe acute respiratory syndrome coronavirus 2"[Title/Abstract]** AND ("Opioid-Related Disorders"[Mesh] OR" Analgesics, Opioid"[Mesh] OR "Narcotics"[Mesh] OR "Narcotic-Related Disorders"[Mesh] OR "Drug Users"[Mesh] OR (((opioid*[Title/Abstract]) OR (opioid addiction[Title/Abstract])) OR (opioid user*[Title/Abstract])) OR (opioid use[Title/Abstract]) AND (english[Filter]))) AND ((((((((((("Emergency Medical Services"[Mesh] OR "Emergency Service, Hospital"[Mesh]) OR "Emergency Medical Technicians"[Mesh]) OR "Emergency Responders"[Mesh]) OR "Inpatients"[Mesh]) OR "Hospitalization"[Mesh]) OR "Community Health Centers"[Mesh]) OR "Community Mental Health Services"[Mesh]) OR "Primary Health Care"[Mesh]) OR "General Practitioners"[Mesh]) OR "Physicians, Family"[Mesh]) OR "Delivery of Health Care"[Mesh] OR (((((((urgent care[Title/Abstract]) OR (hospitalisation[Title/Abstract])) OR (community health centre*[Title/Abstract])) OR (Substance Use Rehabilitation Program*[Title/Abstract])) OR (addition treatment*[Title/Abstract])) OR (substance abuse treatment*[Title/Abstract])) AND (english[Filter]))) AND ((((((((((challenge*[Title/Abstract])) OR (disruption*[Title/Abstract])) OR (impact*[Title/Abstract])) OR (outcome*[Title/Abstract])) OR (cancel*[Title/Abstract])) OR (change*[Title/Abstract])) OR (closure*[Title/Abstract])) OR (access*[Title/Abstract])) OR (patient fear*[Title/Abstract]) OR ((((isolation[Title/Abstract]) OR (social risk factor*[Title/Abstract])) OR (physical distancing[Title/Abstract])) OR (social distancing[Title/Abstract])) OR (healthcare restriction*[Title/Abstract]) OR (social behaviour*[Title/Abstract]) OR (((((((((("Health Facility Closure"[Mesh]) OR "Health Services Accessibility"[Mesh]) OR "Telemedicine"[Mesh]) OR "Infection Control"[Mesh]) OR "Digital Divide"[Mesh]) OR "Internet Access"[Mesh]) OR "Internet"[Mesh]) OR "Fear"[Mesh]) OR "Quarantine"[Mesh]) OR "Social Isolation"[Mesh]) OR "Loneliness"[Mesh] OR "Social Behavior"[Mesh]) | 2 |
|  | "severe acute respiratory syndrome"[MeSH Terms] OR "sars virus"[MeSH Terms] OR "SARS"[Title/Abstract] OR "SARS-Cov"[Title/Abstract] AND ("Opioid-Related Disorders"[Mesh] OR" Analgesics, Opioid"[Mesh] OR "Narcotics"[Mesh] OR "Narcotic-Related Disorders"[Mesh] OR "Drug Users"[Mesh] OR (((opioid*[Title/Abstract]) OR (opioid addiction[Title/Abstract])) OR (opioid user*[Title/Abstract])) OR (opioid use[Title/Abstract]) AND (english[Filter]))) AND ((((((((((("Emergency Medical Services"[Mesh] OR "Emergency Service, Hospital"[Mesh]) OR "Emergency Medical Technicians"[Mesh]) OR "Emergency Responders"[Mesh]) OR "Inpatients"[Mesh]) OR "Hospitalization"[Mesh]) OR "Community Health Centers"[Mesh]) OR "Community Mental Health Services"[Mesh]) OR "Primary Health Care"[Mesh]) OR "General Practitioners"[Mesh]) OR "Physicians, Family"[Mesh]) OR "Delivery of Health Care"[Mesh] OR (((((((urgent care[Title/Abstract]) OR (hospitalisation[Title/Abstract])) OR (community health centre*[Title/Abstract])) OR (Substance Use Rehabilitation Program*[Title/Abstract])) OR (addition treatment*[Title/Abstract])) OR (substance abuse treatment*[Title/Abstract])) AND (english[Filter]))) AND ((((((((((challenge*[Title/Abstract])) OR (disruption*[Title/Abstract])) OR (impact*[Title/Abstract])) OR (outcome*[Title/Abstract])) OR (cancel*[Title/Abstract])) OR (change*[Title/Abstract])) OR (closure*[Title/Abstract])) OR (access*[Title/Abstract])) OR (patient fear*[Title/Abstract]) OR ((((isolation[Title/Abstract]) OR (social risk factor*[Title/Abstract])) OR (physical distancing[Title/Abstract])) OR (social distancing[Title/Abstract])) OR (healthcare restriction*[Title/Abstract]) OR (social behaviour*[Title/Abstract]) OR (((((((((("Health Facility Closure"[Mesh]) OR "Health Services Accessibility"[Mesh]) OR "Telemedicine"[Mesh]) OR "Infection Control"[Mesh]) OR "Digital Divide"[Mesh]) OR "Internet Access"[Mesh]) OR "Internet"[Mesh]) OR "Fear"[Mesh]) OR "Quarantine"[Mesh]) OR "Social Isolation"[Mesh]) OR "Loneliness"[Mesh] OR "Social Behavior"[Mesh]) | 1 |
|  | "Middle East Respiratory Syndrome Coronavirus"[Mesh:NoExp] AND ("Opioid-Related Disorders"[Mesh] OR" Analgesics, Opioid"[Mesh] OR "Narcotics"[Mesh] OR "Narcotic-Related Disorders"[Mesh] OR "Drug Users"[Mesh] OR (((opioid*[Title/Abstract]) OR (opioid addiction[Title/Abstract])) OR (opioid user*[Title/Abstract])) OR (opioid use[Title/Abstract]) AND ((((((((((("Emergency Medical Services"[Mesh] OR "Emergency Service, Hospital"[Mesh]) OR "Emergency Medical Technicians"[Mesh]) OR "Emergency Responders"[Mesh]) OR "Inpatients"[Mesh]) OR "Hospitalization"[Mesh]) OR "Community Health Centers"[Mesh]) OR "Community Mental Health Services"[Mesh]) OR "Primary Health Care"[Mesh]) OR "General Practitioners"[Mesh]) OR "Physicians, Family"[Mesh]) OR "Delivery of Health Care"[Mesh] OR (((((((urgent care[Title/Abstract]) OR (hospitalisation[Title/Abstract])) OR (community health centre*[Title/Abstract])) OR (Substance Use Rehabilitation Program*[Title/Abstract])) OR (addition treatment*[Title/Abstract])) OR (substance abuse treatment*[Title/Abstract])) AND (english[Filter]))) AND ((((((((((challenge*[Title/Abstract])) OR (disruption*[Title/Abstract])) OR (impact*[Title/Abstract])) OR (outcome*[Title/Abstract])) OR (cancel*[Title/Abstract])) OR (change*[Title/Abstract])) OR (closure*[Title/Abstract])) OR (access*[Title/Abstract])) OR (patient fear*[Title/Abstract]) OR ((((isolation[Title/Abstract]) OR (social risk factor*[Title/Abstract])) OR (physical distancing[Title/Abstract])) OR (social distancing[Title/Abstract])) OR (healthcare restriction*[Title/Abstract]) OR (social behaviour*[Title/Abstract]) OR (((((((((("Health Facility Closure"[Mesh]) OR "Health Services Accessibility"[Mesh]) OR "Telemedicine"[Mesh]) OR "Infection Control"[Mesh]) OR "Digital Divide"[Mesh]) OR "Internet Access"[Mesh]) OR "Internet"[Mesh]) OR "Fear"[Mesh]) OR "Quarantine"[Mesh]) OR "Social Isolation"[Mesh]) OR "Loneliness"[Mesh] OR "Social Behavior"[Mesh]) [Limited to 2000-2020] | 7306 |
|  | "Influenza A Virus, H1N1 Subtype"[Mesh:NoExp] AND ("Opioid-Related Disorders"[Mesh] OR" Analgesics, Opioid"[Mesh] OR "Narcotics"[Mesh] OR "Narcotic-Related Disorders"[Mesh] OR "Drug Users"[Mesh] OR (((opioid*[Title/Abstract]) OR (opioid addiction[Title/Abstract])) OR (opioid user*[Title/Abstract])) OR (opioid use[Title/Abstract]) AND ((((((((((("Emergency Medical Services"[Mesh] OR "Emergency Service, Hospital"[Mesh]) OR "Emergency Medical Technicians"[Mesh]) OR "Emergency Responders"[Mesh]) OR "Inpatients"[Mesh]) OR "Hospitalization"[Mesh]) OR "Community Health Centers"[Mesh]) OR "Community Mental Health Services"[Mesh]) OR "Primary Health Care"[Mesh]) OR "General Practitioners"[Mesh]) OR "Physicians, Family"[Mesh]) OR "Delivery of Health Care"[Mesh] OR (((((((urgent care[Title/Abstract]) OR (hospitalisation[Title/Abstract])) OR (community health centre*[Title/Abstract])) OR (Substance Use Rehabilitation Program*[Title/Abstract])) OR (addition treatment*[Title/Abstract])) OR (substance abuse treatment*[Title/Abstract])) AND (english[Filter]))) AND ((((((((((challenge*[Title/Abstract])) OR (disruption*[Title/Abstract])) OR (impact*[Title/Abstract])) OR (outcome*[Title/Abstract])) OR (cancel*[Title/Abstract])) OR (change*[Title/Abstract])) OR (closure*[Title/Abstract])) OR (access*[Title/Abstract])) OR (patient fear*[Title/Abstract]) OR ((((isolation[Title/Abstract]) OR (social risk factor*[Title/Abstract])) OR (physical distancing[Title/Abstract])) OR (social distancing[Title/Abstract])) OR (healthcare restriction*[Title/Abstract]) OR (social behaviour*[Title/Abstract]) OR (((((((((("Health Facility Closure"[Mesh]) OR "Health Services Accessibility"[Mesh]) OR "Telemedicine"[Mesh]) OR "Infection Control"[Mesh]) OR "Digital Divide"[Mesh]) OR "Internet Access"[Mesh]) OR "Internet"[Mesh]) OR "Fear"[Mesh]) OR "Quarantine"[Mesh]) OR "Social Isolation"[Mesh]) OR "Loneliness"[Mesh] OR "Social Behavior"[Mesh]) [Limited to 2000-2020] | 6,629 |
|  | ((("Disease Outbreaks"[Mesh:NoExp]) OR "Natural Disasters"[Mesh]) OR "Disasters"[Mesh:NoExp]) OR "Pandemics"[Mesh:NoExp] AND ("Opioid-Related Disorders"[Mesh] OR" Analgesics, Opioid"[Mesh] OR "Narcotics"[Mesh] OR "Narcotic-Related Disorders"[Mesh] OR "Drug Users"[Mesh] OR (((opioid*[Title/Abstract]) OR (opioid addiction[Title/Abstract])) OR (opioid user*[Title/Abstract])) OR (opioid use[Title/Abstract]) AND ((((((((((("Emergency Medical Services"[Mesh] OR "Emergency Service, Hospital"[Mesh]) OR "Emergency Medical Technicians"[Mesh]) OR "Emergency Responders"[Mesh]) OR "Inpatients"[Mesh]) OR "Hospitalization"[Mesh]) OR "Community Health Centers"[Mesh]) OR "Community Mental Health Services"[Mesh]) OR "Primary Health Care"[Mesh]) OR "General Practitioners"[Mesh]) OR "Physicians, Family"[Mesh]) OR "Delivery of Health Care"[Mesh] OR (((((((urgent care[Title/Abstract]) OR (hospitalisation[Title/Abstract])) OR (community health centre*[Title/Abstract])) OR (Substance Use Rehabilitation Program*[Title/Abstract])) OR (addition treatment*[Title/Abstract])) OR (substance abuse treatment*[Title/Abstract])) AND (english[Filter]))) AND ((((((((((challenge*[Title/Abstract])) OR (disruption*[Title/Abstract])) OR (impact*[Title/Abstract])) OR (outcome*[Title/Abstract])) OR (cancel*[Title/Abstract])) OR (change*[Title/Abstract])) OR (closure*[Title/Abstract])) OR (access*[Title/Abstract])) OR (patient fear*[Title/Abstract]) OR ((((isolation[Title/Abstract]) OR (social risk factor*[Title/Abstract])) OR (physical distancing[Title/Abstract])) OR (social distancing[Title/Abstract])) OR (healthcare restriction*[Title/Abstract]) OR (social behaviour*[Title/Abstract]) OR (((((((((("Health Facility Closure"[Mesh]) OR "Health Services Accessibility"[Mesh]) OR "Telemedicine"[Mesh]) OR "Infection Control"[Mesh]) OR "Digital Divide"[Mesh]) OR "Internet Access"[Mesh]) OR "Internet"[Mesh]) OR "Fear"[Mesh]) OR "Quarantine"[Mesh]) OR "Social Isolation"[Mesh]) OR "Loneliness"[Mesh] OR "Social Behavior"[Mesh]) | 6629 |

**Grey Literature Google Search Terms and Results**

|  | Key words | Results |
| --- | --- | --- |
| Search String 1 | covid 19 OR coronavirus OR pandemic AND "opioid use disorder" OR "substance use disorder" | *102 results (2 open access articles were listed at the top of the results page and included) |
| Search String 2 | covid 19 OR “coronavirus” OR “pandemic”AND "access" AND “healthcare” OR “health care” AND "opioid use disorder" OR "substance use disorder" | 100 |
| Search String 3 | "covid 19" OR “coronavirus” OR “pandemic” AND "opioid use disorder" OR "substance use disorder" AND "primary care" OR “family doctor” OR “family physician” OR “family medicine” | 100 |
| Search String 4 | "covid 19" OR "coronavirus" OR "pandemic" AND "opioid use disorder" OR "substance use disorder" AND "emergency department" OR “emergency room” OR “emergency medicine” | 100 |
| Search String 5 | "covid 19" OR coronavirus OR pandemic AND "opioid use disorder" OR "substance use disorder" AND clinic OR "community health" OR "treatment centre" OR "treatment center" | 100 |
| Search String 6 | covid 19 OR coronavirus OR pandemic AND "opioid addiction" | 100 |
