# Supplementary material for: Opioid use disorder treatment disruptions during the early COVID-19 pandemic and other emergent disasters: a scoping review addressing dual public health emergencies
Source: BMC Public Health. 2021 Jul 28;21:1471. doi: 10.1186/s12889-021-11495-0 (PMC8318046; doi:10.1186/s12889-021-11495-0)
Supplement: Supplementary file 2 — Additional file 2. Supplementary Results of Scientific Literature; this document provides a full list of the included scientific literature and brief overview of sources in a table with descriptions of pertinent findings [file 12889_2021_11495_MOESM2_ESM.docx]

**Additional File 2: Supplementary Results of Scientific Literature**

**TITLE:** Opioid Use Disorder Treatment Disruptions during the COVID-19 Pandemic and Other Disasters: A Scoping Review Addressing Dual Public Health Emergencies

**JOURNAL:** BMC Public Health

**AUTHOR NAMES:** Rita Henderson, Ashley McInnes, Leslee Mackey, Myles Bruised Head, Lindsay Crowshoe, Jessica Hann, Jake Hayward, Brian R. Holroyd, Eddy Lang, Bonnie Larson, Ashley Jane Leonard, Steven Persaud, Khalil Raghavji, Chris Sarin, Hakique Virani, Iskotoahka (William) Wadsworth, Stacey Whitman, Patrick McLane

**CORRESPONDING AUTHOR:** Rita Henderson, Department of Family Medicine, University of Calgary

Email: rihender@ucalgary.ca

**Opioid Use Disorder Care during COVID-19 Scoping Review**

| Citation | Method | Research Participants | Disruption in Care | Healthcare setting | Increased Risk | Risk Mitigation | Cross Systems Issues |
| --- | --- | --- | --- | --- | --- | --- | --- |
| (Agani, Landau, & Agani, 2010) | Model description | N/A | Occupation of Kosovo | General Healthcare systems | The war devastated health and mental health care systems. Pre-war substance use was not an issue, post-war rates of substance use and addiction increased dramatically. | Linking Human Systems (LINC) Community Resilience model was used to develop community-based mental health and addictions services facilitating ties to family, culture and community. Resulted in the establishment of the Kosovo Addiction Treatment, Education and Resource Center. | Before this program there was no addiction treatment or detox support available, only emergency treatment for life threatening situations. International aid organizations that arrived after the war to implement programs that worked in other societies were unsuccessful, as the national trauma required healing specific to rebuilding connections between family, community and culture. |
| (Alexander, Stoller, Haffajee, & Saloner, 2020) | Commentary | N/A | COVID-19 | General Healthcare systems | People with OUD are vulnerable to service disruption as they need frequent interactions with the healthcare system. As many are unstably employed, COVID disruptions to work may lead to loss of housing and food security, increasing risk of relapse | Guidelines allowing take home doses of methadone, remote initiations of controlled substances, reimbursement for telehealth. Recommendation to co-prescribe naloxone to mitigate overdose risk, exempt need for in-person refill of buprenorphine prescription, and provider waivers to increase virtual care, delivery of medications, and wrap around services for those in quarantine. Patient surrogates to pick up and supervise doses may be used, secure pill dispensers or video observed doses may be employed. | During the pandemic the need is great for comprehensive case management with links between substance use disorder treatment, social services, and housing to ensure the safety of people with opioid use disorder. |
| (Arya & Gupta, 2020) | Commentary | N/A | COVID-19 | Addictions treatment | Increased risk of COVID-19 due to comorbid health conditions.  Decreased access to OAT service due to service disruption and lack of public transport. Increased risk of overdose with take home dosing of methadone, as the stress of COVID-19 makes them more likely to use increased dose as a coping strategy. Naloxone is scarcely available and not for take home dosing. Telemedicine not accessible to many Indians. Closure of rehab centres resulted in premature discharge of patients. | Recommendation of take home dosing of methadone and buprenorphine.  Prediction of move to telemedicine. | National organizations need to create strategies to address the needs of people with opioid use disorder during the pandemic. |
| (Banducci & Weiss, 2020) | Commentary | N/A | COVID-19 | Outpatient clinics | People with post-traumatic stress disorder and substance use disorder are particularly vulnerable to the impacts of COVID_19 restrictions, such as social isolation.  Rural and low SES patients may not have access to virtual and telehealth support. | Move to telehealth and virtual support for clients. Increased availability of OAT through longer carriers, telephone renewals, and mailing of medication. Realistic need to focus on harm reduction during pandemic through assessing current substance use and developing plans for safer substance use such as stocking up on supplies, preparing for overdose through having naloxone on hand, and not sharing syringes, pipes or other equipment. | Safety plans for privacy and safety during group telephone session are required.  Safety planning for those who are increasingly at home and may be at risk for domestic violence or suicidal ideation is also necessary. |
| (Basu, Ghosh, Subodh, & Mattoo, 2020) | Commentary | N/A | COVID-19 | Hospital-based OAT outpatient clinic | COVID-19 measures induce stress and withdrawal, which is a potential risk for relapse among individuals with opioid dependence, at increased risk for COVID-19 due to social, housing, living, and medical conditions. | OAT patients will be screened for COVID-19.  Maintain OAT for patients already established on it, no new initiations during COVID-19.  Group counselling moved to virtual platform. | Regulatory mandates for in-person dispensing of buprenorphine; b-n supply challenges; staff shortages |
| (Becker & Fiellin, 2020) | Commentary | N/A | COVID-19 | General health systems | Increase risk of COVID and overdoes during pandemic for people with OUD. serious risk that system-level gains in expanding access to medication for OUD, conducting critical research, and exacting legal reparations against opioid manufacturers will all reverse. We call for urgent action to counteract these risks. | Federal policies relaxed on need for physical exam for methadone prescribing, tele- prescription of buprenorphine now possible. | Call to increase access to OAT through more flexible policies. Call to rapidly expand methadone delivery via mobile teams for quarantined patients. Federal agencies should leverage funds to make OAT more accessible.  Call to ensure that research and legal cases for reparations are not stalled due to the pandemic. |
| (Blake & Lyons, 2016) | Semi-structured interviews | Opioid Treatment program service workers, health professionals, and emergency managers | Disaster Contexts | Opioid Treatment Programs | People with OUD have specific needs that are often not planned for in emergencies. Lack resources such as cell phone credit, Internet access, or transport, make OAT access difficult to organize during disasters. Limited number of providers can prescribe OAT. Multiple barriers to OAT access arise at system level during disasters. Concern that despair plus limited access to OAT leads to illicit drug use. | Calls for collective, multidisciplinary emergency planning for maintenance of OAT during disasters and ensuring the needs of this population are met. Critical to ensure access to OAT stock, prescriptions, and dose verification. Possibility of hospital dispensation of methadone and mobile units discussed. | Training that values cultural specificity and the needs of people who have disabilities, mental health issues, use substances, or are on OAT would prepare frontline responders, organizations, and emergency managers to prepare for disaster scenarios appropriately. |
| (Brown, Melchior, Reback, & Huba, 1994) | Interviews and assessment tool | Women at high risk for HIV infection | Los Angeles Riots | Other- Outreach Workers | No difference in rates of depression or substance use before and after riot were found, but significantly fewer resources were available to substance users post-riot for support for addictions and harm reduction. After the riots there was an increase in homelessness, dependence on welfare, unemployment. Basic needs were hard to meet due to stores being burnt or boarded up. | Outreach workers noticed that women they usually engaged with did not want the usual interactions or were staying inside their homes. Outreach strategy was changed to home visits and provision of basic needs, rather than the usual incentives. | Shortage in treatment services and resources continued at 6 months after the riot. The riot revealed a need for public agencies and community organizations to have partnerships with agencies inside and outside their own community, so that other providers can step in after a disaster. |
| (Columb, Hussain, & O’Gara, 2020) | Commentary- Addiction Psychiatry Service Provision shifts | N/A | COVID-19 | Multiple systems-inpatient and outpatient addictions psychiatry treatment | Risk of relapse with stress, anxiety and social isolation due to the pandemic. Substance use increases vulnerability to COVID-19 and other health impacts. | Reducing number of people in group therapy sessions to enable physical distancing has interrupted group cohesion. Groups became focused on anxiety related to COVID-19, rather than their recovery. Providing equipment to go to tele-psychiatry not possible in short timeframe.  Increased focus on discharge planning and internet support for transition out of treatment facility in climate with decreased support. | Tele-psychiatry should be combined with in person practice after the pandemic ends to provide extended service options. |
| (Cusack, de Crespigny, & Athanasos, 2011) | Discussion paper on literature | N/A | Heatwaves | General healthcare system | Heatwaves increase risk of death and serious illness for people who use substances. May also cause unplanned withdrawal due to inability to access substances. Overextended healthcare systems and power outages increase risk. | Primary and emergency nurses need to educate and support vulnerable clients before and during heatwaves. Heat Safety plans should be made, withdrawal kits should be distributed. | Providers from multiple systems (health care, substance use, mental health, welfare) need to work together to provide adequate care and prevention for vulnerable populations during heat waves and prevent the ambulance and emergency healthcare system from becoming overwhelmed. |
| (Davis & Samuels, 2020) | Commentary | N/A | COVID-19 | Health and justice systems | People with OUD are at increased risk for COVID-19 due to pre-existing health conditions, tobacco use, and likelihood of living in shelters, group recovery housing, or correctional facilities, where physical distancing is difficult. Closures or reduced hours of medical and harm reduction clinics may increase risk of resumed use for people in recovery. Risk of overdose and death is increased by social isolation and overwhelm of emergency response teams. | State provisions to allow for 14 and 28 day take home doses of methadone.  Exemption from in person initiation of buprenorphine, can now be initiated via telehealth.  Some steps to reduce number of people being incarcerated during COVID-19 and release some people from jail.  Police departments have reduce or stopped making arrests for low-level crimes. | All of the changes to increase access to treatment and reduce incarceration during the pandemic should be maintained in perpetuity.  Additionally, States should remove paraphernalia laws that criminalize harm reduction supplies like syringes, provide over the counter naloxone, and crimes of poverty should be responded to with evidence based practices, not incarceration. |
| (Deren, Shedlin, Hamilton, & Hagan, 2002) | Focus Groups, surveys | People who use substances, AIDS outreach workers, service providers | 9/11 | Multiple Healthcare Systems. | Psychological and emotional impacts of 9/11 were reported by substance users. Some reported increased drug use and more open drug use in public immediately after the event, but increases did not sustain. Fears if scare supply and users reported decreased purity of substances after the attack. | Temporary Emergency Medicaid set up after the 9/11 attacks provided medical service to some drug users. AIDS outreach services were not impacted by the crisis. Pharmacies providing syringes was seen as more accessible than local needle exchange facilities. | There was an increase in symptoms of depression and post traumatic stress disorder in residents of New York after the terrorist attacks. Increased police presence was noted by all participants after the attack, but as police focus was on national security their presence was not felt to impact substance users or dealers. |
| (Dunlop et al., 2020) | Commentary | N/A | COVID-19 | Multiple healthcare systems | People with substance use disorders are at Increased risk of COVID-19 due to comorbid health conditions, socioeconomic disadvantage, homelessness or housing instability, imprisonment, and stigma and discrimination in the healthcare community.  Need to access OAT increase risk of COVID-19 transmission. Decrease in harm reduction resources increase risk for HIV and hepatitis. Risk of escalation in substance use due to stress of pandemic. Increased risk of overdose due to isolation changes in drug supply, provision of take home methadone. | Recommendation for provision of longer carries of OAT and take home dosing, along with take home naloxone.  Need to plan for needs of those who need to self-isolate due to COVID-19 exposure, with delivery of medication and home visits, with staff and client safety precautions in place.  Ensuring emergency department staff can efficiently assess and refer people with acute substance use problems to avoid long stays and representations is critical in freeing up emergency resources for COVID-19.  Ensuring harm reduction supplies remain available throughout the pandemic is critical to reduce the risk of blood borne diseases. Vending machine dispensers are an option to reduce in person contact. | People who use substances are already vulnerable in terms of medical, mental and social health and access to support. During the pandemic, providers will be asked to prioritize which of their services are essential and which should be de-prioritized. Services for people who use substances much be configured in the pandemic context. |
| (Elliott, Benoit, Matusow, & Rosenblum, 2017) | Interviews and thematic analysis | OAT providers and directors, State opioid treatment authorities, representatives of the DEA and SAMHSA | Hurricane Sandy and Katrina | Community OAT outpatient clinic | OAT service disruptions (issues with guest dosing, lack of access to regular provider, lack of transportation) put patients and their communities at risk for transmission of infectious diseases and negative consequences associated with relapse (ie. loss of employment, criminal activities. ) Interruptions in OAT services, combined with disruption of illicit drug market increase demand for services at unfamiliar OAT sites and emergency departments. | Recommendations to address risks after future disasters were for Opioid Treatment Programs to universally: 1) educate patients about emergency plans and establish protocol for communication and information sharing between sites during emergencies; 2) provide emergency transportation to  alternate dosing sites; 3) create policies on take home dosing with reciprocity between sites,  4) have backup power  generators; 5) ensure labor provisions for staff overtime  are in place for emergency events; 6) provide all patients with training in overdose response and provide naloxone so that they are prepared in the case that services are disrupted  and 7) conduct regular disaster procedure drills | There are financial and regulatory barriers to implementing the recommendations State Opioid Treatment Authorities indicated they  lack mechanisms to change state policy or the policies of private corporations that would incur the costs of these recommendations. There are serious barriers to strengthening OAT emergency preparedness and a need for greater interagency dialogue. |
| (Farhoudian et al., 2020) | Commentary | N/A | COVID-19 | General Healthcare Systems | Increased risk for COVID and for withdrawal and relapse. Under-housed, lack of access to healthcare, concurrent health disorders | Clinicians are advised to engage family and care providers more than ever during the pandemic. Attracting other sources of social support such as guaranteed wages and an increase in social security payments will improve outcomes | Governments, health authorities, and other relevant stakeholders should make services for substance use disorder essential services |
| (Frank, Dewart, Schmeidler, & Demirjian, 2006) | Interviews | Administrators of Treatment Programs | 9/11 | Drug-free outpatient clinics, Methadone Maintenance clinics, residential treatment programs | Patients and staff were emotionally impacted by the attack and mental health/trauma support was not accessed by most programs. Services were difficult to access given transportation disruptions, particularly for disabled or sick patients. Dosing information for guest dosing of methadone was not verifiable. Increased use of illicit substances was noted. Disaster plans were inadequate to meet the crisis. | Take home doses and guest dosing of methadone at different clinics were offered. Communication issues between patients and service providers were addressed through 24 hour answering services and messages updating program hours. Telephone therapy was important in reaching outpatients who couldn’t get to the clinic. | Drug Free outpatient clinics were more likely to close during the crisis and therefore had subsequent patient retention and financial issues. Federal and State guidelines on dispensing methadone need to be revised. Suggestions to mitigate risks in future involved methadone access through hospitals, mobile vans dispensing methadone, a hotline dedicated to verifying dosage, and the need for database sharing between programs in different parts of the city. |
| (Fried, Liebers, & Roberts, 2020) | Commentary | N/A | COVID-19 | Rural hospitals | Many people with opioid use disorder depend on rural hospitals, which are in danger of closing during COVID-19 due to increased financial strain. | Federal recovery programs are providing short-term aid to rural hospitals during COVID-19. | The pandemic requires that more sustainable funding models for rural hospitals be considered, such as all-payer global budgets. |
| (Galea-Singer et al., 2020) | Commentary | N/A | COVID-19 | Virtual therapy for Substance use | Virtual individual and group sessions decrease risk for COVID-19 but may increase risk that safety and transparency issues are missed, such as suicidal ideation or being on substances during the session. The most vulnerable of the population do not have equitable access to therapy due to not have phones reliable internet. Effectiveness in terms of decreasing substance use is not known. Privacy and confidentiality issues could put people at risk. | Develop protocols around privacy, confidentiality, and procedures to mitigate risks during sessions. Training and supervision for clinicians engaging in virtual therapy is needed. | The pandemic has required innovation to increase accessibility to therapy to all. These innovations will likely remain in place as they improve access for rural populations and others for whom access to in person care is difficult. Research will be necessary to determine the effectiveness of virtual therapy, compared to in person techniques. |
| (Green, Bratberg, & Finnell, 2020) | Commentary | N/A | COVID-19 | Pharmacies | Disruption in OUD care during the pandemic | SAMHSA waived requirement for in person exam to initiate buprenorphine treatment, but not methadone | Pharmacies are considered essential services during the pandemic and have large capacity to increase provision of substance use treatment, education, and education, including both buprenorphine and methadone. |
| (Griffin, Der-Martirosian, Gable, Wyte-Lake, & Dobalian, 2018) | Semi-structured interviews | OAT Executive managers, senior clinicians, administrators | Hurricane Sandy | Inpatient and Outpatient OAT | Due to severe facility damage by the storm, OAT was disrupted much longer than emergency take home and guest dosing allowed for. Guest dosing information could not be efficiently communicated because computers and phones were down. | One day before the storm, several emergency take home doses of methadone were administered to patients. Patients who made it into the clinic after that were given doses and referred to nearby OAT programs. A past OAT site was reopened quickly to serve patients, which is an usual occurrence. | OAT services cannot be easily relocated after a disaster, as each has its own accreditation which is not transferable to another location. OAT programs must stop operating if they cannot reopen by the time emergency guest dosing provisions expire. The process for reopening in a new location is long due to federal, state and local laws. |
| (Harris et al., 2020) | Case Report | OUD patients | COVID-19 | Addictions treatment and street outreach | People with substance use disorder are at increased risk of COVID-19 mortality due to high rates of co-morbid health conditions, being unstably housed and using shelters that have crowding and large numbers of transient people. During COVID-19, interruptions in addiction treatment and harm reduction services mean people with may now be at higher risk for overdose and HIV infection. | Shift to telemedicine for buprenorphine initiation and maintenance. | Tele-buprenorphine initiation offers an important opportunity to lower barriers to OUD treatment and should be delivered in partnership with outreach teams who can deliver harm reduction supplies to highest risk individual who may lack access to telemedicine. |
| (Holloway et al., 2020) | Commentary | N/A | COVID-19 | General healthcare systems | Increased risk due to homelessness, living in shelters, being incarcerated. Decrease in access to harm reduction supplies like naloxone and syringes, disruptions to drug supply chain. | Provision of OAT by telehealth, requires access to internet and cell service. Home delivery of OAT to aid sheltering in place. | Decriminalization of people who use drugs and access to harm reduction must be prioritized during the pandemic. |
| (Jenkins et al., 2020) | Commentary | N/A | COVID-19 | Health, justice and social support systems | People who use substances in rural areas are at increased risk during COVID-19. May lack access to housing and basic necessities for sheltering in place, lack access to healthcare or avoid due to stigma of substance use, take risks to procure drugs that are less available during pandemic. | Supports should be made available for PWUS to have adequate housing situations and resources for quality of life; reduced interactions with the justice system | COVID-19 measures could endanger and further marginalize people who use drugs in rural areas. Lack of access to credible information and pathways to meet CDC guidelines. Risk of withdrawal and health complications due to COVID-19 put stress on the healthcare system, as this population depends on emergency departments for care. Risk of incarceration due to enforcement of social distancing rules |
| (Kar et al., 2020) | Commentary | N/A | COVID-19 | Healthcare systems | Withdrawal, relapse due to missing OAT doses. Development of behavioural addiction | Call to address mental health emergencies such as severe withdrawal symptoms, establish supports for vulnerable community | Need to sensitize people to addictions related issues during pandemic, a mental health helpline may be helpful |
| (Karamouzian, Johnson, & Kerr, 2020) | Commentary | N/A | COVID-19 | General healthcare system | People who use drugs are at increased risk for COVID-19 due to comorbid health conditions, unstable housing, and substance use sharing practices. During COVID-19 they are at increase risk due to reduced access to harm reduction supplies and OAT medication. Overdose risk increases as capacity for emergency response is decreased | Call for increase in harm reduction education and supplies.  Call for communication plan in case of disruption of OAT provision, safe consumption sites and overdose prevention sites.  Call for provision of safe supply to decrease risk of COVID-19 transmission due to need to secure illicit drugs  Call to modify public health messaging for marginalized people who use drugs to focus on minimizing sharing of substance use supplies, emphasizing need to sanitize hands and surfaces and stock up on supplies to reduce trips to harm reduction facilities. Modification of self-isolation message is necessary. | The fundamentals of public health must be used to protect the most vulnerable populations. |
| (Khatri & Perrone, 2020) | Commentary | N/A | COVID-19 | Emergency Department | Emergency department resources are diverted to COVID-19, meaning they are no longer safety net for people with OUD. Most vulnerable patients cannot access telehealth care. Increased risk of overdose due to isolation, risk of mental health worsening and relapse. | Peer recovery team uses texting and video conferencing platforms to do outreach. Phone support is ideal but not accessible to most vulnerable, piloting distributing cell phones to this group. | Disruption in illicit drug supply chain may be opportunity for people to get OUD care. Without safety net of emergency departments, innovative models must be created.  Pharmacies should be recruited during pandemic to serve as sites for naloxone distribution and telemedicine kiosks. |
| (Knopf, 2020a) | Commentary | N/A | COVID-19 | Telehealth | Risk of overdose death due to opioid use. Abrupt discontinuing access to buprenorphine could lead to relapse to substance use, overdoes, and overdose death. | Provision to allow buprenorphine induction by video and telephone, exempting the need for in person interview. Patients with no telephone or who are unstable will still benefit from in-person visits  Assessment of risk for prescribing take home buprenorphine:  Is patient at increased risk for COVID-19? Does patient have ability to safety store take home doses? Who has access in the home (children, pets etc)? How stable is the patient’s opioid use disorder?  one-to-four-week  prescriptions for sublingual  buprenorphine/naloxone  For less stable patients, provide shorter carriers with more frequent video or in person check ins.  Mail order pharmacies to decrease in person contact. | Provisions for access to treatment during COVID-19 are not law and are not permanent. |
| (Knopf, 2020b) | Commentary | N/A | COVID-19 | In-patient treatment centers | Risk of COVID-19 transmission, important to continue cognitive-behavioral intervention for people who use drugs during the stress of the pandemic. | COVID-screening of new patient and visitors, change to virtual care when appropriate |  |
| (Knopf, 2020f) | Commentary | N/A | COVID-19 | General health systems | Possible closure of treatment and recovery organizations increases risk for people who use substances and risk of COVID-19 transmission | Call to increase funding to keep treatment and recovery organizations open, provide PPE for providers, and facilitate change to telehealth | Senators call for $2 billion in supplemental funding for SAMHSA |
| (Knopf, 2020d) | Commentary | N/A | COVID-19 | General health systems | People who use substances at risk due to unemployment and stress of COVID-19. Pandemic exacerbating a shortage of staff and programs for substance use disorder in California. Those in recovery are at high risk for homelessness and relapse. | N/A | Policy makers should respond to the substance use epidemic with the same resources and dedication they are responding to the COVID-19 pandemic. The voice of people who use substances needs to guide response. |
| (Knopf, 2020e) | Commentary | N/A | COVID-19 | OAT outpatient clinic | People with opioid use disorder are at risk of overdose, Hep C, HIV, violence, and COVID-19. | DEA allows initiation of buprenorphine via telehealth consultation. SAMHSA increases methadone carries to 14-28 days. | DEA policy is not a law, but a guideline in place during the pandemic. |
| (Knopf, 2020g) | Commentary | N/A | COVID-19 | OAT outpatient treatment | Risk of COVID-19 transmission and risk of not maintaining OAT. | Recommendations:  No discharge of patients from OUD treatment during COVID-19 unless they endanger their own or staff’s safety.  Administrative detox will be suspended during COVID-19 and dose will not be decreased  Referrals for COVID-19 testing will be made at all Opioid Treatment programs and syringe service sites.  During COVID-19 health care professionals should not be required to complete the training and waiver to prescribe OAT.  Opioid treatment programs, pharmacies and clinicians will work to expand access to Methadone.  Take home dosing privileges should be expanded to whatever extent possible  Telehealth service will replace in person visits.  Requirement for in person methadone initiation should be lifted. Delivery of OAT medications to patients who are isolating due to COVID-19 should be allowable.  State and federal Medicaid should cover cost of take home medications. | National Alliance for Medication  Assisted Recovery  National Alliance for Medication  Assisted Recovery  Recommendations made by the National Alliance for Medication Assisted Recovery were reviewed and sometimes disagreed with by the American Association for the Treatment of Opioid Dependence. |
| (Knopf, 2020c) | Commentary | N/A | COVID-19 | Telephone Buprenorphine | Economic despair, psychiatric  trauma, an inability to access ser-  vices, and a constricting treatment  system in the face of rapid increases  in demand for service  Economic despair, psychiatric  trauma, an inability to access ser-  vices, and a constricting treatment  system in the face of rapid increases  in demand for service  Economic despair, psychiatric  trauma, an inability to access ser-  vices, and a constricting treatment  system in the face of rapid increases  in demand for services paints a pic-  ture that resembles the familiar  graphics which depicted a growing  number of COVID patients and a lim-  ited number of hospital beds and  ventilators, | Provision to allow buprenorphine induction by video and telephone for adolescents, exempting the need for in person interview.  Patients with no telephone or who are unstable will still benefit from in-person visits  Assessment of risk for prescribing take home buprenorphine:  Is patient at increased risk for COVID-19? Does patient have ability to safety store take home doses? Who has access I the home (children, pets etc)? How stable is the patient’s opioid use disorder?  one-to-four-week  prescriptions for sublingual  buprenorphine/naloxone  For less stable patients, provide shorter carriers with more frequent video or in person check ins.  Mail order pharmacies to decrease in person contact. |  |
| (Leppla & Gross, 2020) | Commentary | N/A | COVID-19 | Primary OAT | Rapid shift to virtual practice introduces new risks to patients who benefit from in person accountability. OAT Medication regulations may thwart optimal utilization during COVID  Methadone carriers increase risk of OD, bup\nal may not be adequate for all patients | Virtual dosing, methadone clinic will remain open for highest risk methadone patient. |  |
| (Mackey & Strathdee, 2015) | Commentary | N/A | Multiple disaster contexts | Substance use treatment | Disasters create high risk environments that exacerbate infectious disease spread and drug use. The risk is at personal level of decreased employment, homelessness, discrimination and trauma and the use of substances to cope, as well as macro level of decreases or redirection in public health spending, disruption to treatment services and harm reduction. | Recommendations that global policy makers must (1) proactively identify disasters that may negatively impact substance use and abuse populations; (2) identify factors leading to high risk drug behavior and disease transmission; (3) develop of drug treatment and policy to respond; (4) advocate for need to prioritize substance abuse treatment access in order to prevent disease spread and (5) determine where policy should be directed. | Research is necessary to better understand and address the effects of disasters that exacerbate high risk drug use and infectious disease epidemics. |
| (Marsden et al., 2020) | Commentary | N/A | COVID-19 | Opioid Treatment Programs | People with opioid use disorder at increased risk of COVID-19 due to comorbid health conditions, homelessness, living in shelters, and poverty. Increased risk of overdose due to social isolation and changes in illicit drug supply. Social distancing measures could result in increase in substance use for those who already have problems, relapse for those in recovery, and new onset for those who are vulnerable. | 14 day take home carries of OAT.  Expectation of group and individual therapy by telephone or video. | Information on responding to the needs of people with substance use disorders during the pandemic should be shared internationally and include public health, medical and scientific communities. |
| (McClure, Mendoza, Duncan, Rotrosen, & Hansen, 2014) | Semi-structured Interviews | OAT program providers and administrators | Hurricane Sandy | Hospital-based OAT outpatient clinic | Disruption of OAT due to transportation issues, need to relocate to a different clinic, lack of dosing information, medication supply low at pharmacies. Methadone carries were not long enough to cover disruption in service. | Efforts to maintain access to OAT during disruption included relocating patients to other site, accrediting physicians at new locations, guest dosing. | Lack of clarity around emergency regulations, lack of central database with dosing information, lack of emergency planning between clinics. Patients crowded the local emergency department by turning to them for medication. Some emergency providers refused to provide methadone and some provided potentially dangerous doses of methadone, as dosing information was not available. |
| (Mota, 2020) | Commentary | N/A | COVID-19 | Mental health services | Increased risk due to homelessness, lack of access to healthcare and recovery support, comorbid health conditions and being immunocompromised. | Increased carries of OAT medications to mitigate risk of COVID may increase risk of overdose, with people using in isolation; social and mental health supports are integral components of SU treatment | During pandemic, addictions care must be reinforced, not postponed, so that providers are aware of the additional stress and crises this population may be experiencing in the pandemic. |
| (Movaghar et al., 2005) | Semi-structured interviews and questionnaires | Substance users, families of substance users, healthcare providers, emergency relief workers, people in charge of substance abuse treatment | Bam Earthquake | Multiple healthcare settings | Substance users suffered loss, trauma, and physical injury due to the earthquake. There were no guidelines for prevention and management of drug dependence available for healthcare providers after the earthquake. Most patients experienced withdrawal. A societal increase in the prevalence of drug use is expected post-earthquake due to psychological problems such as posttraumatic stress disorder (PTSD) from the earthquake, as this is a known risk factor for drug abuse. Lack of availability of drugs and increased price risks the shift to more dangerous drugs. | Family and friends brought opium and money to people in hospital who had dependence issues. Some providers took the opportunity to encourage abstaining from drugs. | There were no guidelines for healthcare providers or relief workers on providing care for people who used opioids after the earthquake. Healthcare providers generally had negative attitudes towards patients who used opioids. |
| (O’Dwyer, Cliffe, Watson, McCourt, & Singleton, 2020) | Interviews | Pharmacists and Opioid Treatment Program providers | Hurricane | Opioid Treatment Programs | Noted that disasters interrupt OAT supply and services and therefore increase risk of relapse, risky behaviours, and mortality rates due to overdose.  Storm damage hindered transportation, some services closed due to flooding, and supplies ran low. | Take away dosing of 3-5 days can be issued in an emergency. If service disruption lasts longer than that, patients are referred to emergency departments or other community pharmacy sites, which creates problems because clients lack ID to verify their prescription and dosing, and sometimes dosing information is unavailable if their primary site has closed. | Dosing and prescription information not easily accessible between providers after a disaster.  Some pharmacies ran out of OAT stock and were unaware nearby pharmacies had excess. |
| (Ornell et al., 2020) | Commentary | N/A | COVID-19 | Health systems | Increased risk of overdose, withdrawal, not staying in hospital for care due to substance needs. Risks of COVID-19 due to seeking drug supply, homelessness, incarceration. | Call for training on dealing with substance use disorder for healthcare professionals during the pandemic. | Healthcare professionals must be aware of the risks and challenges when working with people with substance use disorder during COVID-19. |
| (Peavy et al., 2020) | Commentary-Description of Rapid Service Changes | N/A | COVID-19 | Opioid Treatment Programs | OAT reduces risk of HIV by reducing injection drug use, sharing of syringes and equipment, and risk sexual behavior. Therefore during COVID-19, disruption to OAT provision also increases risk of HIV. | Created COVID-19 guidelines with 5 levels of patients according to risk, SAMSHA regulations allowing 14 and 28 day carries depending on stability of patient. Piloted a smartphone app to allow video directly-observed therapy. | This Opioid Treatment Program submitted their clinic guidelines to SAMHSA as a supporting document for the exception on length of carries. This document later became the guideline for all Opioid Treatment clinics in the State. Policy exceptions to ensure flexibility were agreed upon through collaboration between the Health Care Authority and the Governor’s Office and the State Congressional delegation, as well as SAMHSA. |
| (Pouget, Sandoval, Nikolopoulos, & Friedman, 2015) | Interviews | People who Inject Drugs | Hurricane Sandy | Multiple Systems | In the week after the hurricane there was a decrease in supply of illicit drugs, disruptions to harm reduction, OAT, and general health services. Withdrawal was experienced by 60% of study population, 27% reported sharing syringes or equipment and injecting with people they would not normally, and 70% of people on OAT reported not being able to obtain sufficient doses, 43% of HIV-positive participants missed HIV medication doses. Increased risk of HIV transmission and other blood borne diseases | N/A | The needs of people who inject drugs are often overlooked in the context of traumatic and chaotic events. Disruption of services and the illicit drug market resulted in behaviours that increased risk of transmission of HIV and other blood borne diseases. Disruption of OAT services may contribute to the discontinuation of treatment for people who inject drugs. Government agencies, community based organizations, and volunteer agencies should have formal disaster plans that are informed by people who inject drugs. Street services and medical clinics should maintain full stock of syringes in preparation for disasters. |
| (Rogers et al., 2020) | Commentary-Description of rapid service changes | N/A | COVID-19 | Community-based organization | Increased stress and risk during COVID-19 for men living with or at increased risk for HIV who use substances. | Individual sessions continued via telehealth and aimed to manage stress and emotions related to COVID-19 measures. Outreach visits were made to provide harm reduction supplies and Peer Recovery Coach model for psychological support. | The pandemic is shaping healthcare disparities and also behavioural healthcare disparities. The transition to virtual health may widen inequities for vulnerable populations. The task-sharing model of Peer Recovery Coaching, people with lived experience supporting those at risk in less formal ways, was successful in bridging gaps in services. |
| (Ruggiero et al., 2006) | Telephone interviews, online screening questions | NYC residents who experienced 911 | 911 | Internet-based intervention | Disasters increase risk of mental and physical health issues like depression, post-traumatic stress disorder, and increased substance use. | Brief, accessible internet modules were offered to people who had experienced the 9/11 attacks. Modules provide psychoeducation and motivational feedback focused on mental health and substance use issues. Participation and satisfaction rates indicated internet based modules are feasible as disaster aftermath interventions, efficacy was not assessed. | Internet based interventions may increase participation for people who would not seek traditional care, and can be offered rapidly and at low cost. Although internet access in the general population is increasing, it is not available to all. Certain demographics were less likely to participate in the internet intervention, including ethnic minorities and the elderly population. |
| (Rutkow, Vernick, Mojtabai, Rodman, & Kaufmann, 2012) | Commentary | N/A | 911/Hurricane Katrina | Opioid Treatment Programs | Disruption in OAT after a disaster increases risk of withdrawal and relapse. Shelter staff not prepared or trained to meet needs of people who use substances. State and Federal law present barriers to providing service. | There are some Federal and State emergency laws in place to facilitate OAT and program transfers during and after a disaster. SAMHSA issued guidance specific to OAT after Hurricane Katrina noting that clinical judgment could be used when dosing information could not be accessed for displaced patients. | Emergency laws and collaborative agreements for practitioners vary between states and are not consistent for licensing requirements of substance use counselors, creating legal barriers for substance use disorder providers to move between states to meet surges in need. States should act before a disaster to update and clarify emergency laws. Electronic repositories with dosing information should be fully developed. |
| (Salisbury-Afshar, Rich, & Adashi, 2020) | Commentary | N/A | COVID-19 | Health, justice and social support systems | Vulnerable populations at increased risk of COVID-19 and health complications during the pandemic. People with opioid use disorder are at risk of overdose due to drug supply discontinuity, shortage of paraphernalia, social isolation, and loss of income. | SAMHSA emergency regulations waived requirement for in-person exam to initiate buprenorphine, and allow telehealth support for OAT. | Bias against vulnerable populations could increase inequities during COVID-19, such as when ventilators are in short supply and providers are choosing which patient to give it to. Policies and guidelines specific to vulnerable individuals must be in place. |
| (Samuels et al., 2020) | Commentary | N/A | COVID-19 | Addiction treatment and harm reduction sites | Opioid users at increased risk of death due to reduced hours of opioid treatment programs and harm reduction sites. | Telephone initiation of buprenorphine allowed during COVID-19.  Rhode Island Buprenorphine Hotline was initiated to link people with moderate to severe opioid use disorder with providers that can assess and prescribe buprenorphine and then link to outpatient treatment. |  |
| (M. N. Shuler, Wallington, Qualls-Hampton, Podesta, & Suzuki, 2016) | Quantitative analysis of trends in substance use treatment admissions | People admitted to substance use treatment facilities | Hurricane Katrina | Multiple substance use treatment modalities | Homelessness doubled after the hurricane, and is both a cause and a result of substance use. Increased substance use and psychiatric stress were reported post-hurricane, as well as fewer treatment providers and facilities. Patients who did obtain treatment were discharged sooner than ideal, and often placed far from home. | N/A | Louisiana lost 89% of psychiatrists in the state after the hurricane.  Various policy changes directly impacted the number of people admitted for substance use treatment.  A post-hurricane rise in heroin use led to State imposing harsher sentences for dealing in response to this public health issue.  Criminal justice system is the largest source of referrals for substance use treatment in Louisiana. |
| (M. Shuler, Suzuki, Podesta, Qualls-Hampton, & Wallington, 2017) | Quantitative analysis of substance use treatment discharge datasets | People admitted to public and private substance use treatment facilities | Hurricane Katrina | Residential Treatment Centres | Post-hurricane, healthcare and treatment were compromised for those who were evacuated and those who remained in the region. Individuals who had a coexisting psychiatric and substance use disorder had decreased rates of completing treatment for substance use after hurricane Katrina. Unmet treatment needs were associated with homelessness and criminality for those with comorbid psychiatric and substance use issues. | N/A | Financial cuts to mental health and substance use treatment are believed to put those with less severe psychiatric symptoms at greater risk for incarceration, not treatment. |
| (Stowe, Scheibe, Shelly, & Marks, 2020) | Commentary | N/A | COVID-19 | Opioid Treatment Programs and social supports | Increased risk of overdose due to heroin users being placed in shelters to prevent COVID-19 transmission.  Lack of tolerance after period of abstinence puts people at increased risk for overdose and death. Changes in illicit drug supply | Example of one shelter providing methadone to manage withdrawal and one area providing OAT.  Recommendation for government to increase access to OAT in all shelters and link people initiated on OAT during lockdown to ongoing support and maintenance once released. Recommendation to provide overdose education and skills based training during lockdown, and increase access to naloxone. | N/A |
| (Sun et al., 2020) | Commentary | N/A | COVID-19 | Opioid Treatment Programs | People with OUD have comorbid health and mental health conditions, increased risk for COVID-19. Risk due to strain on OAT services during COVID-19 restrictions. Risk of withdrawal, relapse, and overdose increase. | In China, door to door visits and surveys conducted for people with opioid use disorder, to educate them on prevention and screen for COVID-19 symptoms. Delivery of methadone for those located far from clinics. | Four critical actions for authorities to ensure availability of OAT during COVID-19:  Assign staff to deliver methadone and other OAT, comprehensive treatment plans for people with Opioid use disorder who have COVID-19 and are admitted to hospital, provide mental health care for people with opioid use disorder via telephone, vitual platforms or PPE visits, to help with increase in stress and anxiety during COVID-19, and ensure OAT clinics have PPE and are doing COVID-19 screening. More research needed to identify challenges for this population during COVID-19. |
| \| (Tofighi et al., 2014) \| \| --- \| | Surveys administered by phone or in person | OAT buprenorphine patients | Hurricane Sandy | Primary Care | Transit, employment and health system disruption, shortage in buprenorphine after hurricane, financial hardship, loss of housing, prolonged power outages.  Disruption in communication between client and provider and service was limited for 4 weeks. | Providers made unscheduled calls to patients in effort to address disruption in care. Remote telephone prescription refill support , mailing medication, and alternative temporary location providing prescription and medication refill bridged the gap in services for the 4 weeks. | This model of care in which patients are seen weekly or monthly in office while maintaining a supply at home was relatively adaptable after the disaster. Low rates of increased or new onset illicit opioid use were reported, and rates of adherence to buprenorphine treatment were high.  Specific suggestions to address communication breakdown after disasters included: providing a disaster hotlines, access to buprenorphine providers’ mobile phone numbers and emails, and a frequently updated website with disaster-related information. |
| (Tofighi et al., 2015) | Surveys and chart review | OAT buprenorphine patients | Hurricane Sandy | Primary Care | Risk factors for increased substance use after a disaster include psychiatric comorbidity, post-disaster PTSD and depression, and disaster-related losses. | Efforts to bridge the 4 week service gap included telephone contact with patients, calling in refills to open pharmacies, mailing refill prescriptions to stable patients, an obtaining insurance authorizations. | Rates of illicit opioid use remained low 6 months post hurricane and adherence to treatment remained high. Cooperation between government agencies and office based Opioid Treatment programs will reduce risks of relapse and loss of treatment adherence during and after disasters. |
| (Vecchio et al., 2020) | Commentary | N/A | COVID-19 | Opioid Treatment Programs | People with opioid use disorder have increased risk of COVID-19 due to comorbid physical and mental conditions, and increased stigma and marginalization in access to healthcare services.  Provision of take home doses during COVID-19 carriers risk of overdose or diversion of doses. | Change to psychological and social support through video counselling, phone or video used for new patient assessment and triage.  Provision of take home OAT and home delivery of medications. Increased harm reduction supplies.  Limited number of in person visits and strict time slots. Postponement of family therapy. | Prolonged release buprenorphine has been used in other counties and should be made available in Italy as it does not require large amounts of take home medication and is not associated with diversion. |
| (Volkow, 2020) | Commentary | N/A | COVID-19 | Healthcare System | Housing instability, incarceration, reduced access to health care and recovery support services, social isolation and physical distancing, all increase risk of COVID transmission and overdose. | SAMHSA and DEA provide flexibility in OAT prescribing and carries. Call for virtual support meetings and commitment not to marginalize people with substance use disorder. | The health care system, policymakers, and researchers are called upon to innovate ways of meeting the treatment and recovery needs of people with OUD. |
| (Wakeman, Green, & Rich, 2020) | Commentary | N/A | COVID-19 | General healthcare system | People with OUD have increased risk of overdose, isolation and despair due to COVID restrictions, as well as increased risk of COVID-19 | Temporarily waiver of the initial in-person assessment requirement for initiation of buprenorphine and increased flexibility for the dispensation of take-home methadone. treatment remotely. | Call to allow all providers with prescriptive authority to prescribe buprenorphine, and provide emergency funding for buprenorphine and methadone so that all patients can access treatment. Expand ability of pharmacies and post-acute care facilities to prescribe OAT. |
| (Webster, Connoy, Sud, Pinto, & Katz, 2020) | Commentary | N/A | COVID-19 | General healthcare system | COVID-19 safety measures create barriers for accessing pain management and supervised consumption sites. Risk of opioid overdose is increased for people living in chronic pain, with low socioeconomic status or loss of income. This group may not be able to access virtual care. | Health Canada exemption to made controlled substances more available during the pandemic. Providers and organizations made virtual resources available to those dealing with chronic pain. | Policies must intersect to understand and respond appropriately to people living in chronic pain who may also have low socioeconomic status, addictions and mental health issues. We need to better integrate public health, health care, and social services response to serve this vulnerable population during the pandemic. |
| (Weiss et al., 2002) | Semi-structured interviews | Current and former substance users who experience 9/11 | 9/11 | General healthcare system | Post-disaster anxiety, depression and service disruption may lead to increased drug use, including relapse among users who had quit | Generalized fear and anxiety were reported. Mental health professionals, support groups, and counsellors were reported to be available post-9/11 and some felt these supports were the reason why no significant increase in drug use was reported by substance users post 9/11 | N/A |
| (Williams, Tofighi, Rotrosen, Lee, & Grossman, 2014) | Mixed Methods: cross-sectional survey and retrospective chart review | Patients on office-base buprenorphine treatment | Hurricane Sandy | Opioid Treatment program | Major risk factors for increased illicit drug use or relapse post-hurricane were: shorter length of time in treatment, losses due to storm (eg. property damage, evacuation, disruption of buprenorphine supply), a pre-hurricane history of red flag behaviors such as repeat opioid-positive urine tests, and new-onset of psychiatric symptoms post-hurricane. | When clinic shut down, providers met with patients immediately outside of the hospital entrance to provide prescriptions and directions to other facilities. Patients were contacted by phone, and those on Medicaid were mailed their prescriptions. As regular prescriptions of buprenorphine were for 30-90 days, these patients experienced less disruption than others. | Office based buprenorphine is resilient modality in disaster context and should be noted as there is a national shortage of buprenorphine prescribers. Primary care and addictions treatment settings tend to screen out patients with comorbid substance use disorder and psychiatric illness, whereas these patients had good outcomes in this study. |
| (Wilson, Ramage, & Fagan, 2020) | Commentary | N/A | COVID-19 | Primary Care | People with opioid use disorder at increased risk during COVID-19 due to loss of hourly employment, unreliable cell and internet service when services are changing to these modalities and decrease in supports like group therapy. This population also experiences stigma and potential anxiety and depression due to social isolation. People in rural communities may feel impacts of COVID-19 more severely | This rural primary care setting used the following strategy:  All group meetings on hold.  Regular appointments scheduled for 6-8 weeks for low acuity patients and 2-4 weeks for moderate acuity patients, with electronic prescriptions for that length of carry and the option of telehealth visits in the interim. For high acuity patients regular in person visits were maintained. All patients prescribed naloxone. | The pandemic provides the opportunity to reassess how we respond to patient needs and access to services. |

References

Agani, F., Landau, J., & Agani, N. (2010). Community-Building Before, During, and After Times of Trauma: The Application of the LINC Model of Community Resilience in Kosovo. *AMERICAN JOURNAL OF ORTHOPSYCHIATRY*, *80*(1), 143–149. https://doi.org/10.1111/j.1939-0025.2010.01017.x

Alexander, G. C., Stoller, K. B., Haffajee, R. L., & Saloner, B. (2020). An Epidemic in the Midst of a Pandemic: Opioid Use Disorder and COVID-19. *Annals of Internal Medicine*, *173*(1), 57–58. https://doi.org/10.7326/M20-1141

Arya, S., & Gupta, R. (2020). COVID-19 outbreak: Challenges for Addiction services in India. *Asian Journal of Psychiatry*, *51*(March), 102086. https://doi.org/10.1016/j.ajp.2020.102086

Banducci, A. N., & Weiss, N. H. (2020). Caring for Patients With Posttraumatic Stress and Substance Use Disorders During the COVID-19 Pandemic. *Psychological Trauma: Theory, Research, Practice, and Policy*, *12*, 113–114. https://doi.org/10.1037/tra0000824

Basu, D., Ghosh, A., Subodh, B. N., & Mattoo, S. K. (2020). Opioid substitution therapy with buprenorphine-naloxone during COVID-19 outbreak in India: Sharing our experience and interim standard operating procedure. *Indian Journal of Psychiatry*. https://doi.org/10.4103/psychiatry.IndianJPsychiatry_295_20

Becker, W. C., & Fiellin, D. A. (2020). Medications for Opioid Use Disorder Save Lives. *Annals of Internal Medicine*, (April), 59–61. https://doi.org/10.17226/25310

Blake, D., & Lyons, A. (2016). Opioid Substitution Treatment Planning in a Disaster Context: Perspectives from Emergency Management and Health Professionals in Aotearoa/New Zealand. *International Journal of Environmental Research and Public Health*, *13*(11). Retrieved from http://ovidsp.ovid.com/ovidweb.cgi?T=JS&PAGE=reference&D=med13&NEWS=N&AN=27834915

Brown, V. B., Melchior, L. A., Reback, C., & Huba, G. J. (1994). Psychological functioning and substance abuse before and after the 1992 Los Angeles riot in a community sample of women. *Journal of Psychoactive Drugs*, *26*(4), 431–437. https://doi.org/10.1080/02791072.1994.10472463

Columb, D., Hussain, R., & O’Gara, C. (2020). Addiction Psychiatry and COVID-19 - Impact on patients and service provision. *Irish Journal of Psychological Medicine*, 1–15. https://doi.org/10.1017/ipm.2020.47

Cusack, L., de Crespigny, C., & Athanasos, P. (2011). Heatwaves and their impact on people with alcohol, drug and mental health conditions: A discussion paper on clinical practice considerations. *Journal of Advanced Nursing*, *67*(4), 915–922. https://doi.org/10.1111/j.1365-2648.2010.05551.x

Davis, C. S., & Samuels, E. A. (2020). *Opioid Policy Changes During the COVID-19 Pandemic - and Beyond.* Retrieved from https://dx.doi.org/10.1097/ADM.0000000000000679

Deren, S., Shedlin, M., Hamilton, T., & Hagan, H. (2002). Impact of the September 11th attacks in New York City on drug users: a preliminary assessment. *Journal of Urban Health : Bulletin of the New York Academy of Medicine*, *79*(3), 409–412. Retrieved from http://ovidsp.ovid.com/ovidweb.cgi?T=JS&PAGE=reference&D=med4&NEWS=N&AN=12200510

Dunlop, A., Lokuge, B., Masters, D., Sequeira, M., Saul, P., Dunlop, G., … Maher, L. (2020). Challenges in maintaining treatment services for people who use drugs during the COVID-19 pandemic. *Harm Reduction Journal*, *17*(1), 26. https://doi.org/https://dx.doi.org/10.1186/s12954-020-00370-7

Elliott, L., Benoit, E., Matusow, H., & Rosenblum, A. (2017). Disaster preparedness among opioid treatment programs: Policy recommendations from state opioid treatment authorities. *International Journal of Disaster Risk Reduction*, *23*(October 2016), 152–159. https://doi.org/10.1016/j.ijdrr.2017.05.001

Farhoudian, A., Baldacchino, A., Clark, N., Gerra, G., Dom, G., Mokri, A., … Nematollahi, P. (2020). COVID-19 and Substance Use Disorders : Recommendations to a Comprehensive Healthcare Response. *Basic and Clinical Neuroscience*, (April). https://doi.org/10.6084/m9.figshare.12033567

Frank, B., Dewart, T., Schmeidler, J., & Demirjian, A. (2006). The impact of 9/11 on New York City’s substance abuse treatment programs: A study of program administrators. *Journal of Addictive Diseases*, *25*(1), 5–14. https://doi.org/10.1300/J069v25n01_03

Fried, J. E., Liebers, D. T., & Roberts, E. T. (2020). Sustaining Rural Hospitals after COVID-19: The Case for Global Budgets. *JAMA - Journal of the American Medical Association*, *324*(2), 137–138. https://doi.org/10.1001/jama.2020.9744

Galea-Singer, S., Newcombe, D., Farnsworth-Grodd, V., Sheridan, J., Adams, P., & Walker, N. (2020). Challenges of virtual talking therapies for substance misuse in New Zealand during the COVID-19 pandemic: an opinion piece. *The New Zealand Medical Journal*, *133*(1515), 104–111.

Green, T. C., Bratberg, J., & Finnell, D. S. (2020). Opioid use disorder and the COVID 19 pandemic: A call to sustain regulatory easements and further expand access to treatment. *Substance Abuse*, *41*(2), 147–149. https://doi.org/10.1080/08897077.2020.1752351

Griffin, A. R., Der-Martirosian, C., Gable, A., Wyte-Lake, T., & Dobalian, A. (2018). A Crisis Within a Crisis: The Extended Closure of an Opioid Treatment Program After Hurricane Sandy. *JOURNAL OF DRUG ISSUES*, *48*(4), 536–545. https://doi.org/10.1177/0022042618779541

Harris, M., Johnson, S., Mackin, S., Saitz, R., Walley, A. Y., & Taylor, J. L. (2020). *Low Barrier Tele-Buprenorphine in the Time of COVID-19: A Case Report.* Retrieved from https://dx.doi.org/10.1097/ADM.0000000000000682

Holloway, I. W., C Spaulding, A., Miyashita Ochoa, A., A Randall, L., R King, A., & Frew, P. M. (2020). COVID-19 vulnerability among people who use drugs: recommendations for global public health programmes and policies. *Journal of the International AIDS Society*, *23*(7), 23–25. https://doi.org/10.1002/jia2.25551

Jenkins, W. D., Bolinski, R., Bresett, J., Van Ham, B., Fletcher, S., Walters, S., … Ouellet, L. (2020). COVID-19 During the Opioid Epidemic – Exacerbation of Stigma and Vulnerabilities. *Journal of Rural Health*, *00*, 1–3. https://doi.org/10.1111/jrh.12442

Kar, S. K., Arafat, S. M. Y., Sharma, P., Dixit, A., Marthoenis, M., & Kabir, R. (2020). COVID-19 pandemic and addiction: Current problems and future concerns. *Asian Journal of Psychiatry*, *51*(102064).

Karamouzian, M., Johnson, C., & Kerr, T. (2020). Public health messaging and harm reduction in the time of COVID-19. *The Lancet Psychiatry*, *7*(5), 390–391. https://doi.org/10.1016/S2215-0366(20)30144-9

Khatri, U. G., & Perrone, J. (2020). Opioid Use Disorder and COVID-19: Crashing of the Crises. *Journal of Addiction Medicine*, *14*(4). https://doi.org/10.1097/ADM.0000000000000684

Knopf, A. (2020a). ASAM on buprenorphine inductions by telephone. *Alcoholism & Drug Abuse Weekly*, *32*(16), 4–6. https://doi.org/10.1002/adaw.32694

Knopf, A. (2020b). ASAM recommends virtual treatment of SUD for some patients. *Alcoholism & Drug Abuse Weekly*, *32*(15), 4–5. https://doi.org/10.1002/adaw.32687

Knopf, A. (2020c). Buprenorphine for adolescents via telephone. *Brown University Child & Adolescent Behavior Letter*, *36*(6), 9–10. https://doi.org/10.1002/cbl.30473

Knopf, A. (2020d). CCAPP: Number of deaths from untreated addiction may rival those from COVID‐19. *Alcoholism & Drug Abuse Weekly*, *32*(21), 1–5. https://doi.org/10.1002/adaw.32729

Knopf, A. (2020e). DEA allows buprenorphine inductions based on telephone only. *Alcoholism & Drug Abuse Weekly*, *32*(14), 4–5. https://doi.org/10.1002/adaw.32680

Knopf, A. (2020f). Senators call for $2 billion in supplemental SAMHSA funding. *Alcoholism &amp; Drug Abuse Weekly*. https://doi.org/10.1002/adaw.32738

Knopf, A. (2020g). Sign‐on letter requests more flexibility in methadone treatment during COVID‐19. *Alcoholism & Drug Abuse Weekly*, *32*(15), 1–4. https://doi.org/10.1002/adaw.32685

Leppla, I. E., & Gross, M. S. (2020). Optimizing Medication Treatment of Opioid Use Disorder During COVID-19 (SARS-CoV-2). *Journal of Addiction Medicine*, *14*(4), 19–21. https://doi.org/10.1097/ADM.0000000000000678

Mackey, T. K., & Strathdee, S. A. (2015). Big events and risks to global substance using populations: Unique threats and common challenges. *Substance Use and Misuse*, *50*(7), 885–890. https://doi.org/10.3109/10826084.2015.983008

Marsden, J., Darke, S., Hall, W., Hickman, M., Holmes, J., Humphreys, K., … West, R. (2020). Mitigating and learning from the impact of COVID-19 infection on addictive disorders. *Addiction*, *115*(6), 1007–1010. https://doi.org/10.1111/add.15080

McClure, B., Mendoza, S., Duncan, L., Rotrosen, J., & Hansen, H. (2014). Effects of Regulation on Methadone and Buprenorphine Provision in the Wake of Hurricane Sandy. *JOURNAL OF URBAN HEALTH-BULLETIN OF THE NEW YORK ACADEMY OF MEDICINE*, *91*(5), 999–1008. https://doi.org/10.1007/s11524-014-9904-5

Mota, P. (2020). Avoiding a new epidemic during a pandemic: The importance of assessing the risk of substance use disorders in the COVID-19 era. *Psychiatry Research*, *290*(May), 113142. https://doi.org/10.1016/j.psychres.2020.113142

Movaghar, A. R., Goodarzi, R. R., Izadian, E., Mohammadi, M. R., Hosseini, M., & Vazirian, M. (2005). The impact of Bam earthquake on substance users in the first 2 weeks: A rapid assessment. *JOURNAL OF URBAN HEALTH-BULLETIN OF THE NEW YORK ACADEMY OF MEDICINE*, *82*(3), 370–377. https://doi.org/10.1093/jurban/jti089

O’Dwyer, N., Cliffe, H., Watson, K. E., McCourt, E., & Singleton, J. A. (2020). Continuation of opioid replacement program delivery in the aftermath of cyclones in Queensland, Australia: A qualitative exploration of the perspectives of pharmacists and opioid replacement therapy staff. *Research in Social and Administrative Pharmacy*, *16*(8), 1081–1086. https://doi.org/10.1016/j.sapharm.2019.11.007

Ornell, F., Moura, H. F., Scherer, J. N., Pechansky, F., Kessler, F. H. P., & von Diemen, L. (2020). The COVID-19 pandemic and its impact on substance use: Implications for prevention and treatment. *Psychiatry Research*, *289*(May), 113096. https://doi.org/10.1016/j.psychres.2020.113096

Peavy, K. M., Darnton, J., Grekin, P., Russo, M., Green, C. J. B., Merrill, J. O., … Tsui, J. I. (2020). Rapid Implementation of Service Delivery Changes to Mitigate COVID-19 and Maintain Access to Methadone Among Persons with and at High-Risk for HIV in an Opioid Treatment Program. *AIDS Behav*. https://doi.org/10.1007/s10461-020-02887-1

Pouget, E. R., Sandoval, M., Nikolopoulos, G. K., & Friedman, S. R. (2015). Immediate impact of Hurricane Sandy on people who inject drugs in New York City. *Substance Use and Misuse*, *50*(7), 878–884. https://doi.org/10.3109/10826084.2015.978675

Rogers, B. G., Arnold, T., Schierberl Scherr, A., Strong, S. H., Holcomb, R., Daley Ndoye, C., … Chan, P. A. (2020). Adapting Substance Use Treatment for HIV Affected Communities During COVID-19: Comparisons Between a Sexually Transmitted Infections (STI) Clinic and a Local Community Based Organization. *AIDS and Behavior*, *24*(11), 2999–3002. https://doi.org/10.1007/s10461-020-02933-y

Ruggiero, K. J., Resnick, H. S., Acierno, R., Carpenter, M. J., Kilpatrick, D. G., Coffey, S. F., … Galea, S. (2006). Internet-based intervention for mental health and substance use problems in disaster-affected populations: A pilot feasibility study. *BEHAVIOR THERAPY*, *37*(2), 190–205. https://doi.org/10.1016/j.beth.2005.12.001

Rutkow, L., Vernick, J. S., Mojtabai, R., Rodman, S. O., & Kaufmann, C. N. (2012). Legal challenges for substance abuse treatment during disasters. *Psychiatric Services*, *63*(1), 7–9. https://doi.org/10.1176/appi.ps.2012000p7

Salisbury-Afshar, E. M., Rich, J. D., & Adashi, E. Y. (2020). Vulnerable Populations: Weathering the Pandemic Storm. *American Journal of Preventive Medicine*, *58*(6), 892–894. https://doi.org/10.1016/j.amepre.2020.04.002

Samuels, E. A., Clark, S. A., Wunsch, C., Keeler, L. A. J., Reddy, N., Vanjani, R., & Wightman, R. S. (2020). *Innovation During COVID-19: Improving Addiction Treatment Access.* Retrieved from https://dx.doi.org/10.1097/ADM.0000000000000685

Shuler, M. N., Wallington, S. F., Qualls-Hampton, R. Y., Podesta, A. E., & Suzuki, S. (2016). Trend Analysis of Substance Abuse Treatment Admissions in New Orleans From 2000-2012: A Population-Based Comparison Pre- and Post-Hurricane Katrina. *SUBSTANCE USE & MISUSE*, *51*(12), 1542–1554. https://doi.org/10.1080/10826084.2016.1188952

Shuler, M., Suzuki, S., Podesta, A., Qualls-Hampton, R., & Wallington, S. F. (2017). A Post-Hurricane Katrina Examination of Substance Abuse Treatment Discharges With Co-Occurring Psychiatric and Substance Use Disorders. *Journal of Dual Diagnosis*, *13*(2), 144–156. https://doi.org/10.1080/15504263.2016.1277816

Stowe, M.-J., Scheibe, A., Shelly, S., & Marks, M. (2020). COVID-19 restrictions and increased risk of overdose for street-based people with opioid dependence in South Africa. *South African Medical Journal / Suid-Afrikaanse Tydskrif Vir Geneeskunde*, *110*(6). https://doi.org/10.7196/SAMJ.2020.v110i6.14832

Sun, Y., Bao, Y., Kosten, T., Strang, J., Shi, J., & Lu, L. (2020). Editorial: Challenges to Opioid Use Disorders During COVID-19. *Am J Addict*. https://doi.org/10.1111/ajad.13031

Tofighi, B., Grossman, E., Goldfeld, K. S., Williams, A. R., Rotrosen, J., & Lee, J. D. (2015). Psychiatric comorbidity and substance use outcomes in an office-based buprenorphine program six months following hurricane sandy. *Substance Use and Misuse*, *50*(12), 1571–1578. https://doi.org/10.3109/10826084.2015.1023455

Tofighi, B., Grossman, E., Williams, A. R., Biary, R., Rotrosen, J., & Lee, J. D. (2014). Outcomes among buprenorphine-naloxone primary care patients after Hurricane Sandy. *Addiction Science & Clinical Practice*, *9*, 3. https://doi.org/https://dx.doi.org/10.1186/1940-0640-9-3

Vecchio, S., Ramella, R., Drago, A., Carraro, D., Littlewood, R., & Somaini, L. (2020). COVID19 pandemic and people with opioid use disorder: innovation to reduce risk. *Psychiatry Research*. https://doi.org/10.1016/j.psychres.2020.113047

Volkow, N. D. (2020). Collision of the COVID-19 and Addiction Epidemics. *Annals of Internal Medicine*, *173*(1), 61–62. https://doi.org/10.7326/M20-1212

Wakeman, S. E., Green, T. C., & Rich, J. (2020). An overdose surge will compound the COVID-19 pandemic if urgent action is not taken. *Nature Medicine*, *26*(June), 819–820. https://doi.org/10.1038/s41591-020-0889-1

Webster, F., Connoy, L., Sud, A., Pinto, A. D., & Katz, J. (2020). Grappling with Chronic Pain and Poverty during the COVID-19 Pandemic. *Canadian Journal of Pain*, *4*(1), 125–128. https://doi.org/10.1080/24740527.2020.1766855

Weiss, L., Fabri, A., McCoy, K., Coffin, P., Netherland, J., & Finkelstein, R. (2002). A vulnerable population in a time of crisis: Drug users and the attacks on the World Trade Center. *JOURNAL OF URBAN HEALTH-BULLETIN OF THE NEW YORK ACADEMY OF MEDICINE*, *79*(3), 392–403. https://doi.org/10.1093/jurban/79.3.392

Williams, A. R., Tofighi, B., Rotrosen, J., Lee, J. D., & Grossman, E. (2014). Psychiatric Comorbidity, Red Flag Behaviors, and Associated Outcomes among Office-Based Buprenorphine Patients Following Hurricane Sandy. *JOURNAL OF URBAN HEALTH-BULLETIN OF THE NEW YORK ACADEMY OF MEDICINE*, *91*(2), 366–375. https://doi.org/10.1007/s11524-014-9866-7

Wilson, C. G., Ramage, M., & Fagan, E. B. (2020). A Primary Care Response to COVID-19 for Patients with an Opioid Use Disorder. *Journal of Rural Health*, *00*, 1–3. https://doi.org/10.1111/jrh.12438
