# Supplementary material for: Opioid use disorder treatment disruptions during the early COVID-19 pandemic and other emergent disasters: a scoping review addressing dual public health emergencies
Source: BMC Public Health. 2021 Jul 28;21:1471. doi: 10.1186/s12889-021-11495-0 (PMC8318046; doi:10.1186/s12889-021-11495-0)
Supplement: Supplementary file 3 — Additional file 3. Supplementary Results of Grey Literature, this document provides a brief summary of the grey literature, highlighting critical documents [file 12889_2021_11495_MOESM3_ESM.docx]

**Additional File 3: Supplementary Results of Grey Literature**

**TITLE:** Opioid Use Disorder Treatment Disruptions during the COVID-19 Pandemic and Other Disasters: A Scoping Review Addressing Dual Public Health Emergencies

**JOURNAL:** BMC Public Health

**AUTHOR NAMES:** Rita Henderson, Ashley McInnes, Leslee Mackey, Myles Bruised Head, Lindsay Crowshoe, Jessica Hann, Jake Hayward, Brian R. Holroyd, Eddy Lang, Bonnie Larson, Ashley Jane Leonard, Steven Persaud, Khalil Raghavji, Chris Sarin, Hakique Virani, Iskotoahka (William) Wadsworth, Stacey Whitman, Patrick McLane

**CORRESPONDING AUTHOR:** Rita Henderson, Department of Family Medicine, University of Calgary

Email: rihender@ucalgary.ca

There were four results that stood out as critical to disseminating policy changes impacting the treatment of opioid use disorder (OUD) in Canada during COVID-19 (see table 1 for a summary of results by source type).

**Brief Summary of Grey literature**

*Health Canada toolkit: COVID-19 and substance use* (Government of Canada, 2020)

Federally, the “Health Canada toolkit: COVID-19 and substance use” result provided clear information to help providers understand their options in supporting people who use drugs (PWUD) during the pandemic, and help PWUD understand what services may be available to them. The toolkit information identified three ways PWUD are at increased risk during COVID-19:

- - Increase risk of overdose and harm due to increasingly toxic and unpredictable illegal drug supply
  - Increase risk of withdrawal due to need to self-isolate or quarantine
  - Increase risk of infection due to underlying health conditions

The toolkit included links to the information on:

- Exemptions to the Controlled Drugs and Substances Act during COVID-19,
- Frequently asked questions about legislative and regulatory requirements for substance use disorder (SUD) treatment and providing of safer supply during COVID-19, including federal requirements and when to refer to province-specific requirements, and
- Drug plan coverage for medications used for treatment and safe supply provision for SUD.

The Health Canada toolkit also linked to the three additional resources identified in our search results: CRISM’s National Response documents, BCCSU’s Risk Mitigation in the Context of Dual health Emergencies document, and BCCSU’s COVID-19: Information for Opioid Agonist Treatment Prescribers and Pharmacists.

*The Centre for Research in Substance Misuse (CRISM) National Rapid Response Documents* (CRISM, 2020e, 2020d, 2020b, 2020c, 2020a, 2020f)

The Centre for Research in Substance Misuse (CRISM) had six results. They brought together leaders in the field of Substance Use treatment and created a series of six National Rapid Response documents for responding to COVID-19. The National Rapid Response documents stood out for their comprehensiveness, each ranging from 37 to 82 pages long. Three of these documents focused on harm reduction and safe supply for people with SUD living in shelters, in hospital due to COVID-19, and in residential and supportive recovery programs. Three more CRISM documents focused on the safety of Harm Reduction workers during COVID-19, telemedicine guidelines, and medications and other strategies to support physical distancing during COVID-19.

*Risk Mitigation in the Context of Dual Health Emergencies (BC Centre on Substance Use* (BCCSU, 2020b)

BCCSU was active in posting news releases, links to news articles, and links to Guidelines and policy updates during the pandemic. Two results stood out as critical resources (and were also included as links on Health Canada’s toolkit site). The first is the” Risk Mitigation in the Context of Dual health Emergencies” document, 25 pages long, and published in March of 2020. The document was intended to guide providers in supporting people who use substances to self-isolate or social distance to prevent the spread of COVID-19. The document advocates for replacing the illicit drug supply with prescribed and regulated substances on a daily basis, to allow people who use street drugs and are at high risk for COVID-19 infection (or who have a confirmed or suspected case of COVID-19) to prevent withdrawal, self-isolate, and decrease the risk of COVID-19.

*COVID-19: Information for Opioid Agonist Treatment Prescribers and Pharmacists* (BCCSU, 2020a)

The second high impact result from BCCSU is “COVID-19: Information for Opioid Agonist Treatment Prescribers and Pharmacists. This document was first published on March 17, 2020 and outlined guidelines for increasing carries, reducing time of witnessed doses, and adjusting opioid agonist treatment (OAT) medications according to risk assessment.

*OAT Prescribing Practice Changes* (Health Canada, 2020)

OAT prescribing practice guidelines was the most frequent topic in our results, with 14 results focusing on prescribing practice during COVID-19 and the exemptions made for controlled substances for prescribers, patients, and pharmacists (often re-iterations of Health Canada’s Subsection 56(1) class exemption). Health Canada’s Subsection 56(1) class exemption for patients, practitioners and pharmacists prescribing and providing controlled substances in Canada during the coronavirus pandemic. The exemption allows:

- pharmacists to extend and renew prescriptions,
- pharmacists to transfer prescriptions to other pharmacists, within the same province,
- practitioners to verbally prescribe prescriptions with controlled substances; and
- individuals (e.g., pharmacy technicians, pharmacy employee, courier or individual requested by the patient, etc.) to deliver controlled substances to patients (at their homes or an alternate location)

The resulting changes in OAT protocol for patients included:

- Decrease or no in-person visits, switch to virtual appointments
- No daily witnessed dosing
- No urine tests required
- Increased carries of Buprenorphine/Naloxone (bup/nal) and Methadone
- For patients on injectable OAT, consider transition to oral opioids and providing carries

*Summary of other results: Provincial responses, clinic closures and practice changes*

Four Provincial government website sections were found on substance use or opioid use disorder treatment and resources during COVID, primarily focused on OAT during COVID-19, with one providing information on Mental Health during COVID-19 that included tips for those dealing with SUD, and one with specific direction for those seeking help for OUD (Government of New Brunswick, 2020; Government of NFLD, 2020a, 2020c, 2020b).

Four results were from Regional Health authorities (Peterborough Public Health, 2020; Vancouver Coastal Health, 2020a, 2020b; Windsor-Essex County Health Unit, 2020). Peterborough Public Health had a webpage on Mental Health and Substance Use during COVID-19, providing tips and links to the numbers for a Rapid Access Addiction Medicine clinic and a resource for Naloxone kits. Vancouver Coastal Health had one page on guidelines for overdose prevention during COVID-19 and one with an information sheet to help prescribers to aid people who use substances manage withdrawal and encourage self-isolation during COVID-19.

Nine results were community addiction clinics websites (including municipal websites or community programs with information on addiction clinics) with information on any service changes during COVID-19 (Addictions Services of Thames Valley, 2020; ConnexOntario, 2020; Homeless Hub, 2020; Lakeridge Health, 2020; Providence Healthcare, 2020; Red Book, 2020; Renascent, 2020; Thunder Bay District Health Unit, 2020; Women’s College Hospital, 2020). These outlined clinic closures as well as changes to by-appointment only (no walk-ins) or to virtual appointments. Some clinics had no change in service but new hygiene protocols.

Four results regarded residential addictions treatment and indicated the centres had all stayed open during COVID-19, with new protocols in place for reducing the risk of COVID (Addiction Rehab Toronto, 2020; Aurora Addictions Treatment Centre, 2020; Halton Community Services Directory, 2020; Toronto Addiction Rehab Centre, 2020). Two of those sites advocated that the COVID-19 crisis was an ideal time for loved ones to enter rehab.

Table 1: Summary of Results by Source Type

| Source Type | Number of Results | Location/Organization |
| --- | --- | --- |
| Health Canada | 5 | Canada |
| Provincial government website | 4 | New Brunswick (1), Newfoundland (3) |
| Provincial Colleges of Pharmacy and pharmacy networks | 7 | Alberta(2), Saskatchewan(1), Newfoundland(2), Ontario (1), CATP (1) |
| BC Centre for Substance Use (BCCSU) | 7 | BC, many results relevant nationally |
| Addiction medicine networks and societies | 12 | CRISM, CSAM, CAMH, CCSA, The Royal MH Care and Research |
| Regional Health Units | 4 | Vancouver Coastal Health, Peterborough Public Health, Windsor-Essex Health Unit |
| Addiction clinics or programs | 9 | Vancouver, Thunder bay, Hamilton, Halton/Mississauga, Peterborough, Oshawa, Thames Valley, CATC clinics across Ontario. |
| Residential Treatment Programs | 4 | Ontario |
| News Articles | 15 | CTV, CBC, Reuters, Global, Thorold News, Calgary Herald , National Post, The Tyee |
| First Nations Health Authority (BC) | 4 | BC |
| BC Centre for Disease Control | 1 | BC |
| Total | 72 |  |

**References**

Addiction Rehab Toronto. (2020). How We Are Helping to Treat Addiction During the COVID-19 Pandemic. Retrieved October 8, 2020, from https://addictionrehabtoronto.ca/helping-treat-addiction-covid-19-pandemic/

Addictions Services of Thames Valley. (2020). Information regarding COVID 19.

Aurora Addictions Treatment Centre. (2020). COVID-19, Addiction Services & Accessible Testing: A Letter to Doug Ford & Christine Elliott. Retrieved October 8, 2020, from https://www.freedomaddiction.ca/blog/covid-19-letter-to-gov/

BCCSU. (2020a). COVID-19: Information for Opioid Agonist Treatment Prescribers and Pharmacists 2020. Retrieved September 14, 2020, from https://www.bccsu.ca/wp-content/uploads/2020/05/COVID-19-Bulletin-May-20-2020.pdf

BCCSU. (2020b). Risk Mitigation in the Context of Dual Public Health Emergencies: Interim Clinical Guidance. Retrieved September 14, 2020, from https://www.bccsu.ca/wp-content/uploads/2020/04/Risk-Mitigation-in-the-Context-of-Dual-Public-Health-Emergencies-v1.5.pdf

Canadian Society of Addiction Medicine. (2020). A Harm Reduction Approach to Managing Opioid Use Disorder During COVID-19: A Brief Summary for Clinicians 2020 Mar 28. Retrieved September 14, 2020, from https://csam-smca.org/wp-content/uploads/2020/03/SummaryofOUDCOVID19_2.pdf

ConnexOntario. (2020). Addiction, Mental Health, and Problem Gambling Treatment Services.

CRISM. (2020a). Harm reduction worker safety during the COVID-19 global pandemic.

CRISM. (2020b). Medications and other clinical approaches to support physical distancing for people who use substances during the COVID-19. Retrieved October 8, 2020, from https://crism.ca/wp-content/uploads/2020/06/CRISM-Guidance-Medications-and-other-clinical-approaches-22062020-final.pdf

CRISM. (2020c). Strategies to Reduce SARS-CoV-2 Transmission in Supportive Recovery Programs and Residential Addiction.

CRISM. (2020d). Supporting people who use substances in acute care settings during the COVID-19 pandemic. Retrieved October 8, 2020, from https://crism.ca/wp-content/uploads/2020/06/Supporting-People-Who-Use-Substances-in-Acute-Care-Settings-during-the-COVID-19-Pandemic-CRISM-Interim-Guidance-Document-12062020.pdf

CRISM. (2020e). Supporting people who use substances in shelter settings during the COVID‑19 pandemic: national rapid guidance. Retrieved October 8, 2020, from https://crism.ca/wp-content/uploads/2020/06/CRISM-Guidance-Supporting-People-Who-Use-Substances-in-Emergency-Shelter-Settings-V1.pdf

CRISM. (2020f). Telemedicine support for addiction services.

Government of Canada. (2020). Health Canada toolkit: COVID-19 and substance use. Retrieved October 8, 2020, from https://www.canada.ca/en/health-canada/services/substance-use/toolkit-substance-use-covid-19.html

Government of New Brunswick. (2020). Methadone for the Treatment of Opioid Use Disorder. Retrieved October 8, 2020, from https://www2.gnb.ca/content/gnb/en/departments/health/MedicarePrescriptionDrugPlan/methadone_opioid_dependence.html

Government of NFLD. (2020a). Mental Health and Wellness.

Government of NFLD. (2020b). Opioid Agonist Treatment (OAT) Guidance During the COVID-19 Pandemic March 31, 2020.

Government of NFLD. (2020c). Supporting People Requiring Opioid Dependence Treatment. Retrieved October 8, 2020, from https://www.gov.nl.ca/covid-19/resources/supporting-people-requiring-opioid-dependence-treatment/

Halton Community Services Directory. (2020). St. Joseph’s Healthcare Hamilton.

Health Canada. (2020). Subsection 56(1) class exemption for patients, practitioners and pharmacists prescribing and providing controlled substances in Canada during the coronavirus pandemic 2020 Mar 19. Retrieved September 14, 2020, from https://www.canada.ca/en/health-canada/services/health-concerns/controlled-substances-precursor-chemicals/policy-regulations/policy-documents/section-56-1-class-exemption-patients-pharmacists-practitioners-controlled-substances-covid-19-pandemic.html

Homeless Hub. (2020). Supporting Survivors’ Access to Substance Use Disorder and Mental Health Services During the COVID-19 Emergency. Retrieved October 8, 2020, from https://www.homelesshub.ca/resource/supporting-survivors’-access-substance-use-disorder-and-mental-health-services-during-covid

Lakeridge Health. (2020). Rapid Access Addiction Medicine (RAAM).

Peterborough Public Health. (2020). Novel Coronavirus (COVID-19) – Mental Health & Substance Use. Retrieved October 8, 2020, from https://www.peterboroughpublichealth.ca/for-professionals/health-professionals/novel-coronavirus-2019-ncov-health-professionals/novel-coronavirus-covid-19-mental-health-substance-use/

Providence Healthcare. (2020). Rapid Access Addiction Clinic (RAAC). Retrieved October 8, 2020, from http://www.providencehealthcare.org/rapid-access-addiction-clinic-raac

Red Book. (2020). St. Joseph’s Healthcare Hamilton.

Renascent. (2020). 12-step Meetings in a Time of Coronavirus.

Thunder Bay District Health Unit. (2020). COVID-19: People Who Use Substances.

Toronto Addiction Rehab Centre. (2020). ROAD TO RECOVERY THROUGH COVID-19. Retrieved October 8, 2020, from https://renascent.ca/wp-content/uploads/2020/04/Road-to-Recovery-Through-COVID-19.pdf

Vancouver Coastal Health. (2020a). Overdose prevention and response during Covid-19.

Vancouver Coastal Health. (2020b). Prescriber Guidelines for Risk Mitigation in the Context of Dual Public Health Emergencies Notification to all prescribers in the Vancouver Coastal Health (VCH) region.

Windsor-Essex County Health Unit. (2020). News Release: May 21st Public Health Updates Related to Coronavirus (COVID-19). Retrieved October 8, 2020, from https://www.wechu.org/newsroom/news-release-may-21st-public-health-updates-related-coronavirus-covid-19

Women’s College Hospital. (2020). Substance Use Service. Retrieved October 8, 2020, from https://www.womenscollegehospital.ca/care-programs/substance-use-service
