# Supplementary material for: Opioid use disorder treatment disruptions during the early COVID-19 pandemic and other emergent disasters: a scoping review addressing dual public health emergencies
Source: BMC Public Health. 2021 Jul 28;21:1471. doi: 10.1186/s12889-021-11495-0 (PMC8318046; doi:10.1186/s12889-021-11495-0)
Supplement: Supplementary file 4 — Additional file 4. Literature Summary Sheet, this document provides infographics synthesizing the academic and grey literature according to three thematic areas (increased risk, risk mitigation, and cross-systems issues) that guided the NGT discussions [file 12889_2021_11495_MOESM4_ESM.pdf]

LITERATURE SUMMARY SHEET

CIHR IRSC
Canadian Institutes of Health Research | Instituts de recherche en santé du Canada

UNIVERSITY OF CALGARY

UNIVERSITY OF ALBERTA

OPIOID USE DISORDER DURING COVID-19

THEME:
People with OUD are at increased risk during disasters/pandemics

People with opioid use disorder (OUD) are at increased risk of negative health outcomes during the COVID-19 pandemic due to co-existing medical and mental health conditions, unstable housing, and reduced access to addiction treatment, recovery supports, and harm reduction services (including naloxone and safe consumption sites). Dependence on the informal economy for income and the need to seek illicit drugs puts people with OUD at increased risk for COVID-19 transmission. They are also at increased risk for overdose due to increasing toxicity of illicit drug supply and using alone due to social isolation and COVID-19 safety measures.

Increased risk of Overdose (OD)

- Reports of illicit drug supply becoming more toxic/new and unfamiliar products coming into circulation, increasing the risk of overdose (OD). (1-7)
- Decrease in access to safe consumption sites and increase of drug use in isolation increases risk of OD.(4, 5, 9)
- Some providers see federal income support payments as increasing risk of OD during COVID-19.(10)
- Decrease in access and availability of naloxone during COVID-19(4), fear of transmission through nasal naloxone may result in less OD rescue.(5)
- During COVID-19, lack of access to PPE means greater general reluctance to respond to OD with rescue breaths. There have been calls for Personal Protective Equipment as harm reduction.(5)

Increased risk of Relapse

- Loss of income, social isolation, increase in stress, anxiety and fear were reported to increase risk of using illicit drugs for people with substance use disorder after 9/11, hurricanes Sandy and Katrina, and during COVID-19.(3, 4, 8, 11)
- Lack of access to treatment, peer support groups and recovery support increase risk for relapse. (4, 5, 8, 11)
- Withdrawal due to disruptions in access to OAT is a risk factor for returning to illicit supply.(8, 12)
- Rural and remote communities have increased barriers to OUD care.(13)

Increased Risk of Covid-19

People with OUD:

- Commonly have coexisting health conditions or are immunosuppressed, making them vulnerable to COVID-19 infection.(7, 14, 15)
- Are more often under-housed or living in shelters or incarcerated, making social distancing difficult. (7, 14-16)
- May need to be interacting socially due to dependence on the informal economy for money, seeking illegal drug supply, or seeking OAT. (5, 15)
- Are at risk for not self-isolating or remaining in hospital for COVID-19 treatment if OUD needs are not met. (17)

Lessons from Previous Disaster Contexts

- After Hurricane Sandy, a study showed those who experienced either interrupted OAT supply or who sustained property loss or evacuation as a result of the hurricane were more likely to increase illicit substance use or relapse.(8)
- Mental distress in the aftermath of 9/11 left many OAT patients confused and displaying extremes in emotions. Providers noted freque return to illicit drug use.(11)
- After a cyclone in Australia, patients on OAT were unable to access their regular dosing sites and were rerouted to emergency departments. Treatment was hampered by lack of communication between providers on dosing and previous prescriptions. Legal restrictions requiring ID or other information were barriers to treatment. Relapse risk increased during service disruption.(12)

References – Increased Risk

1. Browne R. Calls for safer opioid supply grow as COVID-19 pandemic compounds overdose crisis Online News Article: Global News; 2020, Mar 20 [Available from: https://globalnews.ca/news/6706851/opioid-supply-coronavirus/.
2. Uguen-Csenge E. B.C. releases plan to provide safe supply of drugs during COVID-19 pandemic Online News Article: CBC; 2020 Mar 26 [Available from: https://www.cbc.ca/news/canada/british-columbia/safe-supply-drug-plan-covid-1.5511973.
3. Wakeman S, Green T, Rich J. An overdose surge will compound the COVID-19 pandemic if urgent action is not taken. Nature Medicine. 2020 May 1;26:819-20.
4. CCSA. CCENDU Alert: Changes Related to COVID-19 in the Illegal Drug Supply and Access to Services, and Resulting Health Harms: Canadian Centre on Substance Use and Addiction; 2020 [Available from: https://www.ccsa.ca/sites/default/files/2020-05/CCSA-COVID-19-CCENDU-Illegal-Drug-Supply-Alert-2020-en.pdf.
5. CCSA. Impacts of the COVID-19 Pandemic on People Who Use Substances: What We Heard. Candian Centre on Substance Use and Addiction,2020 [Available from: https://www.ccsa.ca/sites/default/files/2020-07/CCSA-COVID-19-Impacts-on-People-Who-Use-Substances-Report-2020-en.pdf.
6. Takeuchi C. As B.C. reports record number of overdose deaths, Dr. Bonnie Henry makes emotional plea for help Online Article: Georgia Straight; 2020 Jun 12 [Available from: https://www.straight.com/living/as-bc-reports-record-number-of-overdose-deaths-dr-bonnie-henry-makes-emotional-plea-for-help.
7. Volkow ND. Collision of the COVID-19 and Addiction Epidemics. Ann Intern Med. 2020;173(1):61-2.
8. Williams AR, Tofighi B, Rotrosen J, Lee JD, Grossman E. Psychiatric comorbidity, red flag behaviors, and associated outcomes among office-based buprenorphine patients following Hurricane Sandy. J Urban Health. 2014;91(2):366-75.
9. Bains C. Coroner to feds: Take action on B.C. overdose epidemic like with pandemic. Online Article: National Post; 2020 Jun 11 [Available from: https://nationalpost.com/pmn/news-pmn/canada-news-pmn/cp-newsalert-b-c-records-highest-overdose-death-toll-for-one-month-in-may.
10. Cullen C. CERB benefits contributing to spike in overdoses, outreach workers warn Online Article: CBC; 2020 Jun 10 [Available from: https://www.cbc.ca/news/politics/cerb-pandemic-opioid-addiction-overdose-1.5606188.
11. Frank B, Dewart T, Schmeidler J, Demirjian A. The impact of 9/11 on New York City's substance abuse treatment programs: a study of program administrators. J Addict Dis. 2006;25(1):5-14.
12. O'Dwyer N, Cliffe H, Watson KE, McCourt E, Singleton JA. Continuation of opioid replacement program delivery in the aftermath of cyclones in Queensland, Australia: A qualitative exploration of the perspectives of pharmacists and opioid replacement therapy staff. Res Social Adm Pharm. 2020;16(8):1081-6.
13. BC Centre for Substance Use. Risk Mitigation in the Context of Dual Public Health Emergencies: Interim Clinical Guidance 2020 Mar [Available from: https://www.bccsu.ca/wp-content/uploads/2020/04/Risk-Mitigation-in-the-Context-of-Dual-Public-Health-Emergencies-v1.5.pdf.
14. Canadian Society of Addiction Medicine. A Harm Reduction Approach to Managing Opioid Use Disorder During COVID-19: A Brief Summary for Clinicians 2020 Mar 28 [Available from: https://csam-smca.org/wp-content/uploads/2020/03/SummaryofOUDCOVID19_2.pdf.
15. Karamouzian M, Johnson C, Kerr T. Public health messaging and harm reduction in the time of COVID-19. Lancet Psychiatry. 2020;7(5):390-1.
16. Davis CS, Samuels EA. Opioid Policy Changes During the COVID-19 Pandemic - and Beyond. J Addict Med. 2020;14(4):e4-e5.
17. Dong K, Meador K, Hyshka E, Salokangas E, BurtonMacLeod S, Bablitz C, et al. Supporting People Who Use Substances in Acute Care Settings during the COVID-19 Pandemic: CRISM - Interim Guidance Document. Version 1. Edmonton, AB: Canadian Research Initiative in Substance Misuse; 2020 Jun 9 [Available from: https://crism.ca/wp-content/uploads/2020/06/Supporting-People-Who-Use-Substances-in-Acute-Care-Settings-during-the-COVID-19-Pandemic-CRISM-Interim-Guidance-Document-12062020.pdf.

LITERATURE SUMMARY SHEET

OPIOID USE DISORDER DURING COVID-19

THEME: Efforts to Mitigate Risk for People with OUD During COVID-19

People with opioid use disorder (OUD) are at increased risk of overdose, relapse, and COVID-19 infection. Service disruption caused by shelter in place mandates, physical distancing, and the closure or reduced hours of many services for people with OUD increases those risks. Multiple policy and service-based changes were made to increase accessibility to Opioid Agonist Therapy (OAT) and safe supply. Due to what is being called an overdose epidemic, advocates are calling for decriminalization and regulation of the illicit drug market.

Disruption to Service

- Social distancing and shelter in place measures to limit COVID-19 spread affect accessibility of safe consumption sites, community clinics and doctor's offices.(1-3)
- Public perception is that emergency departments (EDs) should be avoided. There have been public health messages for people with OUD to not go to ED unless seriously ill during COVID-19. (4)
- Many clinics that provide OAT stopped accepting walk ins, some closed.(5, 6)
- Peer support groups and counselling closed or changed to phone or virtual meetings, not accessible to all.(2, 7)

Efforts to Mitigate Risk

- Calls were made to focus on harm reduction and increase access to safe supply during COVID. Clinical Guideline documents on prescribing safe supply were released from BCCSU and CRISM (8-20)
- Health Canada exemption made OAT prescribing more flexible(21).
- Some services changed to telephone or virtual appointments/meetings.(22, 23)
- OAT can now be initiated by telephone or virtual visit at provider's discretion.(21, 23)
- Longer carries and removing requirement for urine tests and witnessed dosing decreased need for in-person visits.(23)
- Verbal transfers of prescriptions between pharmacies, delivery of OAT by pharmacy, and family/friends able to pick up OAT address access issues.(21, 23)

Outcomes

- Support for physicians newly prescribing OAT through guideline documents.(20)
- BC implemented a 24/7 provider support line for OAT guidance.(24)
- Prescription of safe supply increased by 150% in BC during the pandemic(11)
- Jan-May 2020, 89 overdose deaths reported among First Nations people in BC, nearly double 2019 number. (18)
- July-August 2020: BC OUD providers call for decriminalization and federal regulation of illicit drug market to stop overdose crisis.(18, 25, 26)
- Calls have been made for increased flexibility in OAT prescribing to be maintained post-pandemic.(27, 28)

LESSONS FROM PREVIOUS DISASTERS

- Based on experiences after Hurricanes Sandy and Katrina, disaster mitigation for Opioid Treatment Programs should include: improved patient-provider communication in order to relay changes in procedures, providing transportation to patients, allowing for take-home dosing, ensuring all patients have access to Naloxone (and are educated on usage).
- Multiple US studies recognized that telephone and virtual care solutions may be inaccessible to under-housed and low income OUD patients, and provision of phones or mobile support may be necessary to mitigate lack of access to phones and computers.

References – Risk Mitigation

1. Bains C. Coroner to feds: Take action on B.C. overdose epidemic like with pandemic. Online Article: National Post; 2020 Jun 11 [Available from: https://nationalpost.com/pmn/news-pmn/canada-news-pmn/cp-newsalert-b-c-records-highest-overdose-death-toll-for-one-month-in-may.
2. Windsor-Essex County Health Unit News Release: May 21st Public Health Updates Related to Coronavirus (COVID-19) 2020 [Available from: https://www.wechu.org/newsroom/news-release-may-21st-public-health-updates-related-coronavirus-covid-19.
3. Volkow ND. Collision of the COVID-19 and Addiction Epidemics. Ann Intern Med. 2020;173(1):61-2.
4. Government of Newfoundland and Labrador. Supporting People Requiring Opioid Dependence Treatment 2020 [Available from: https://www.gov.nl.ca/covid-19/resources/supporting-people-requiring-opioid-dependence-treatment/.
5. St Joseph's Healthcare Hamilton. Womankind Addiction Service : Temporarily Suspended until July 2020 Online Directory: Halton Community Services Directory; 2020 [Available from: https://halton.cioc.ca/record/OAK4142.
6. Lakeridge Health Rapid Access Addiction Medicine. COVID-19 Service Related Changes 2020 [Available from: https://www.lakeridgehealth.on.ca/en/ourservices/rapid-access-addiction-medicine--raam-.asp.
7. The Royal Mental Health Care and Research. COVID-19 Q&A: Coping with a substance use disorder 2020 [Available from: https://www.theroyal.ca/news/covid-19-qa-coping-substance-use-disorder.
8. Karamouzian M, Johnson C, Kerr T. Public health messaging and harm reduction in the time of COVID-19. Lancet Psychiatry. 2020;7(5):390-1.
9. Winter J. Drug Users Call for Safe Supply of Heroin and Cocaine, and Show How It's Done Online Article: The Tyee; 2020 Jun 23 [Available from: https://thetyee.ca/News/2020/06/23/Safe-Supply-Drug-Demo/.
10. Browne R. Calls for safer opioid supply grow as COVID-19 pandemic compounds overdose crisis Online News Article: Global News; 2020, Mar 20 [Available from: https://globalnews.ca/news/6706851/opioid-supply-coronavirus/.
11. Takeuchi C. As B.C. reports record number of overdose deaths, Dr. Bonnie Henry makes emotional plea for help Online Article: Georgia Straight; 2020 Jun 12 [Available from: https://www.straight.com/living/as-bc-reports-record-number-of-overdose-deaths-dr-bonnie-henry-makes-emotional-plea-for-help.
12. Elton-Marshall T, Ali F, Hyshka E, Shahin R, Hopkins S, Imtiaz S, et al. Harm reduction worker safety during the COVID-19 global pandemic: National Rapid Guidance. Version 1. Toronto, ON: Canadian Research Initiative in Substance Misuse; 2020 Jul 6 [Available from: https://crism.ca/wp-content/uploads/2020/07/CRISM-Guidance-Worker-Safety-06072020.pdf.
13. Brar R, Bruneau J, Butt P, Goyer M, Lim R, Poulin G, et al. Medications and other clinical approaches to support physical distancing for people who use substances during the COVID-19 pandemic: National Rapid Guidance Document. Version 1.
Vancouver, BC: Canadian Research Initiative in Substance Misuse; 2020 Jun 22 [Available from: https://crism.ca/wp-content/uploads/2020/06/CRISM-Guidance-Medications-and-other-clinical-approaches-22062020-final.pdf.
14. Cooksey J, Ganci G, McPherson C, Plant B, Resch K, Robinson S, et al. Strategies to Reduce SARS-CoV-2 Transmission in Supportive Recovery Programs and Residential Addiction Treatment Services: National Rapid Guidance Document. Version 1. Vancouver, BC: Canadian Research Initiative in Substance Misuse; 2020 Jun 30 [Available from: https://crism.ca/wp-content/uploads/2020/06/Strategies-to-Reduce-SARS-CoV-2-Transmission-in-Supportive-Recovery-Programs-and-Residential-Addiction-Treatment-Services-30062020.pdf.
15. Dong K, Meador K, Hyshka E, Salokangas E, BurtonMacLeod S, Bablitz C, et al. Supporting People Who Use Substances in Acute Care Settings during the COVID-19 Pandemic: CRISM - Interim Guidance Document. Version 1. Edmonton, AB: Canadian Research Initiative in Substance Misuse; 2020 Jun 9 [Available from: https://crism.ca/wp-content/uploads/2020/06/Supporting-People-Who-Use-Substances-in-Acute-Care-Settings-during-the-COVID-19-Pandemic-CRISM-Interim-Guidance-Document-12062020.pdf.
16. Hyshka K, Dong K, Meador K, Speed K, Abele B, LeBlanc S, et al. Supporting people who use substances in shelter settings during the COVID-19 pandemic. Version 1. Edmonton, Alberta: Canadian Research Initiative in

Substance Misuse; 2020 May 17 [Available from: https://crism.ca/wp-content/uploads/2020/06/CRISM-Guidance-Supporting-People-Who-Use-Substances-in-Emergency-Shelter-Settings-V1.pdf.
17. Bruneau J, Rehm J, Wild TC, Wood E, Sako A, Swansburg J, et al. Telemedicine Support for Addiction Services: National Rapid Guidance Document. Version 1. Montreal, Quebec: Canadian Research Initiative in Substance Misuse. 2020 May 15 [Available from: https://crism.ca/wp-content/uploads/2020/05/CRISM-National-Rapid-Guidance-Telemedicine-V1.pdf.
18. Wyton M. June Overdose Toll Hits Record; 15 Times More Deadly than COVID-19 Online Article: The Tyee; 2020 Jul 16 [Available from: https://thetyee.ca/News/2020/07/16/June-Overdose-Toll/.
19. Uguen-Csenge E. B.C. releases plan to provide safe supply of drugs during COVID-19 pandemic Online News Article: CBC; 2020 Mar 26 [Available from: https://www.cbc.ca/news/canada/british-columbia/safe-supply-drug-plan-covid-1.5511973.
20. BC Centre for Substance Use. Risk Mitigation in the Context of Dual Public Health Emergencies: Interim Clinical Guidance 2020 Mar [Available from: https://www.bccsu.ca/wp-content/uploads/2020/04/Risk-Mitigation-in-the-Context-of-Dual-Public-Health-Emergencies-v1.5.pdf.
21. Health Canada. Subsection 56(1) class exemption for patients, practitioners and pharmacists prescribing and providing controlled substances in Canada during the coronavirus pandemic 2020 Mar 19 [Available from: https://www.canada.ca/en/health-canada/services/health-concerns/controlled-substances-precursor-chemicals/policy-regulations/policy-documents/section-56-1-class-exemption-patients-pharmacists-practitioners-controlled-substances-covid-19-pandemic.html.
22. Rogers BG, Arnold T, Schierberl Scherr A, Strong SH, Holcomb R, Daley Ndoye C, et al. Adapting Substance Use Treatment for HIV Affected Communities During COVID-19: Comparisons Between a Sexually Transmitted Infections (STI) Clinic and a Local Community Based Organization. AIDS Behav. 2020:1-4.
23. BC Centre for Substance Use. COVID-19: Information for Opioid Agonist Treatment Prescribers and Pharmacists 2020 [Available from: https://www.bccsu.ca/wp-content/uploads/2020/05/COVID-19-Bulletin-May-20-2020.pdf.
24. BC Centre for Substance. BC launches 24/7 helpline to help medical professionals care for people with addictions 2020 Jun 16 [Available from: https://www.bccsu.ca/blog/news-release/bc-launches-24-7-helpline-to-help-medical-professionals-care-for-people-with-addictions/.
25. Fellicella G. Record B.C. overdose deaths mean it's time to listen Online Article: Gerogia Strait; 2020 Jul 17 [Available from: https://www.straight.com/news/record-bc-overdose-deaths-mean-its-time-to-listen.
26. Bambury B. Amid 'horrific' spike in opioid deaths, doctors call for decriminalization, regulation of drugs Online Article: CBC radio; 2020 Jul 24 [Available from: https://www.cbc.ca/radio/day6/portland-protests-how-covid-affects-weather-forecasts-canada-s-opioid-crisis-love-on-the-spectrum-and-more-1.5660707/amid-horrific-spike-in-opioid-deaths-doctors-call-for-decriminalization-regulation-of-drugs-1.5660720.
27. Davis CS, Samuels EA. Opioid Policy Changes During the COVID-19 Pandemic - and Beyond. J Addict Med. 2020;14(4):e4-e5.
28. Green TC, Bratberg J, Finnell DS. Opioid use disorder and the COVID 19 pandemic: A call to sustain regulatory easements and further expand access to treatment. Subst Abus. 2020;41(2):147-9.

LITERATURE SUMMARY SHEET

CIHR IRSC
Canadian Institutes of Health Research / Instituts de recherche en santé du Canada

UNIVERSITY OF CALGARY

UNIVERSITY OF ALBERTA

OPIOID USE DISORDER DURING COVID-19

THEME: Cross systems issues affecting care for people with OUD

Research after past disasters discussed cross systems issues affecting care for people with OUD. The US hurricane literature calls for well defined emergency procedures including stronger professional networks and cross-coverage plans, flexibility around regulations, and a central registry with patient prescription information.(1). Ability to meet the Opioid Agonist Therapy (OAT) needs of people who are ill or disabled were noted as a gap that must be addressed (2,3). There is substantial literature on efforts to ensure prescribers are working with pharmacies to make OAT accessible. There are calls for substance use disorder treatment services to be integrated with housing and social services and acute care to help ensure the safety of patients with OUD.(4-6) Innovations in care in response to service disruptions are also reported, such as mobile clinics, hotlines for patients and prescribers, distribution of phones and virtual care access sites.

Cross Systems issues with OUD Care	Examples from literature
Disruption of pharmaceutical supply chain: Potential that manufacture and transport of OAT is disrupted.	• An interstate travel ban in India during COVID-19 restricted transport of OAT because OAT medications were not listed as essential medications and thus not exempt from the ban.(7)<br>• In Australia after a cyclone, accessible clinics ran out of OAT supply and were not aware that nearby pharmacies had excess.(8)
Regulatory agencies make exemptions and provisions to maintain OAT: Delays in communication between regulators and providers, inadequate regulatory changes to meet needs during and after disaster.	• There have been communication from regulatory agencies around policy changes not getting to providers in time causing delays and disruption in OAT care.(1)<br>• After US hurricanes, regulated carries were not long enough to cover service disruption, pharmacists and administrators felt pressure to “bend the rules” to maintain OAT.(1,3)<br>• Hurricane literature cites long regulatory processes for new OAT clinics that hampered care resumption when physical sites were destroyed.(1)<br>• In Canada and US OAT prescription and dispensing were made flexible for both buprenorphine/naloxone and methadone.(3,9,10)<br>• US Federal regulations on need for physical exam for methadone prescribing were relaxed during COVID-19 and longer carries for stable patients were allowed. To help reduce ED crowding, there have been calls to eliminate federal training requirements for buprenorphine/naloxone and allow emergency medicine clinicians without waivers to write buprenorphine/naloxone prescriptions at discharge.(9)
Disruption to OAT initiation/Call for Safe Supply: Disruption of care at OAT sites requires other physicians and sites to respond to OUD for maintenance and provision of safe supply.	• In the US, hurricane research indicated that some emergency medicine providers were reluctant to prescribe methadone to patients who were unable to access their regular site.(11)<br>• Concern that during COVID-19 emergency medicine providers with increased caseloads would be less likely to initiate buprenorphine/naloxone.(12)<br>• In the US, poor access to illicit opioid supply may be an opportunity for people to start OAT, call to scale up availability of OAT and outreach efforts to encourage people.(13)<br>• During COVID-19, India made provision for telehealth but no provision to initiate OAT via telehealth, resulting in policies of no initiations, except through emergency visits due to overdose or withdrawal.(7)<br>• In Canada CRISM released documents to inform shelter providers, acute care providers, and general physicians about OUD needs/safe supply in these settings.(5,6,14)<br>• There have been calls to make naloxone more available as people are not accessing their usual harm reduction sites.(15)

THEME: Cross systems issues affecting care for people with OUD

Cross Systems issues with OUD Care	Examples from literature
Clinics, pharmacies, and service providers shifting care models to fill in gaps in OUD services and address overdose crisis	• Following Hurricane Sandy, a mobile methadone unit was successfully deployed in collaboration between local, state and federal agencies.(16) Mobile OAT clinics have been recommended during COVID-19 to aid sheltering in place and quarantining.(9)<br>• During COVID-19, BC started a 24/7 provider support line for physicians new to treating people with SUD (17) and released the Lifeguard App for opioid users that automatically connects them to First Responders if they become unresponsive.(18)<br>• BC Centre for Substance use reported that in one week in June, over 3000 prescribers and pharmacists received webinar training in prescribing safe supply. (19)<br>• BC announced $4.27 million in permanent funding during COVID-19 to expand substance use integrated health teams of nurses, counsellors, social workers, Indigenous support workers and outreach staff with the goal of connecting people with treatment, addressing housing issues, supporting COVID-19 related issues.(20)<br>• The Rhode Island Buprenorphine Hotline was created to fill gaps of traditional OAT clinics that operate only during business hours and limited walk-ins due to COVID-19. The 24 hour Hotline operates as a tele-bridge clinic that connects patients with care providers who can do an initial assessment and, if appropriate, prescribe buprenorphine/naloxone for unwitnessed induction and connection to outpatient treatment.(21)
Switch to Virtual Care via video or telephone: addresses access to care for rural populations or those with transportation challenges and allows social distancing during COVID-19, but exacerbates inequity in health care access for those without phones and internet who are already at increased risk	• OAT maintenance, addictions counselling, recovery support, peer group meetings, and changes to virtual or telephone formats during COVID-19 raise concerns of lack of access to cell phones and internet, privacy and confidentiality issues, and missing serious risk factors like suicidal ideation due to increased group support rather than individual check ins.(22)<br>• Providers need immediate guidelines and training on conducting virtual therapy and addressing limitations of this modality.(22,23)<br>• Access to care inequities noted for marginalized groups, including those living with poverty, under-housed persons, sex workers, sexual minorities, and people at increased risk for or living with HIV.(24)
Public Health Messages not always appropriate for people with OUD: providers recommend modifying emergency response public health messages to reduce harm for people with OUD	• COVID-19 public health messages to shelter in place and keep physical distance is in opposition to the long-standing harm reduction model for people with OUD to never use alone and to seek help quickly for overdose and withdrawal.(19,25)<br>• There have been calls for modifying public health messages around self-isolation and physical distancing for people who use drugs who live in shelters or who are involved in sex work during COVID-19.(25)

References – Cross Systems Issues

1. McClure B, Mendoza S, Duncan L, Rotrosen J, Hansen H. Effects of regulation on methadone and buprenorphine provision in the wake of Hurricane Sandy. J Urban Health. 2014;91(5):999-1008.
2. Frank B, Dewart T, Schmeidler J, Demirjian A. The impact of 9/11 on New York City's substance abuse treatment programs: a study of program administrators. J Addict Dis. 2006;25(1):5-14.
3. Griffin AR, Der-Martirosian C, Gable A, Wyte-Lake T, Dobalian A. A Crisis Within a Crisis: The Extended Closure of an Opioid Treatment Program After Hurricane Sandy. Journal of Drug Issues. 2018;48(4):536-45.
4. Alexander GC, Stoller KB, Haffajee RL, Saloner B. An Epidemic in the Midst of a Pandemic: Opioid Use Disorder and COVID-19. Ann Intern Med. 2020;173(1):57-8.
5. Dong K, Meador K, Hyshka E, Salokangas E, BurtonMacLeod S, Bablitz C, et al. Supporting People Who Use Substances in Acute Care Settings during the COVID-19 Pandemic: CRISM - Interim Guidance Document. Version 1. Edmonton, AB: Canadian Research Initiative in Substance Misuse; 2020 Jun 9 [Available from: https://crism.ca/wp-content/uploads/2020/06/Supporting-People-Who-Use-Substances-in-Acute-Care-Settings-during-the-COVID-19-Pandemic-CRISM-Interim-Guidance-Document-12062020.pdf.
6. Hyshka K, Dong K, Meador K, Speed K, Abele B, LeBlanc S, et al. Supporting people who use substances in shelter settings during the COVID-19 pandemic. Version 1. Edmonton, Alberta: Canadian Research Initiative in Substance Misuse; 2020 May 17 [Available from: https://crism.ca/wp-content/uploads/2020/06/CRISM-Guidance-Supporting-People-Who-Use-Substances-in-Emergency-Shelter-Settings-V1.pdf.
7. Basu D, Ghosh A, Subodh BN, Mattoo SK. Opioid substitution therapy with buprenorphine-naloxone during COVID-19 outbreak in India: Sharing our experience and interim standard operating procedure. Indian J Psychiatry. 2020;62(3):322-6.
8. O'Dwyer N, Cliffe H, Watson KE, McCourt E, Singleton JA. Continuation of opioid replacement program delivery in the aftermath of cyclones in Queensland, Australia: A qualitative exploration of the perspectives of pharmacists and opioid replacement therapy staff. Res Social Adm Pharm. 2020;16(8):1081-6.
9. Becker WC, Fiellin DA. When Epidemics Collide: Coronavirus Disease 2019 (COVID-19) and the Opioid Crisis. Annals of internal medicine. 2020;173(1):59-60.
10. Health Canada. Subsection 56(1) class exemption for patients, practitioners and pharmacists prescribing and providing controlled substances in Canada during the coronavirus pandemic 2020 Mar 19 [Available from: https://www.canada.ca/en/health-canada/services/health-concerns/controlled-substances-precursor-chemicals/policy-regulations/policy-documents/section-56-1-class-exemption-patients-pharmacists-practitioners-controlled-substances-covid-19-pandemic.html.
11. Elliott L, Benoit E, Matusow H, Rosenblum A. Disaster Preparedness among Opioid Treatment Programs: Policy Recommendations from State Opioid Treatment Authorities. International Journal of Disaster Risk Reduction. 2017;23.
12. Volkow ND. Collision of the COVID-19 and Addiction Epidemics. Ann Intern Med. 2020;173(1):61-2.
13. Wakeman S, Green T, Rich J. An overdose surge will compound the COVID-19 pandemic if urgent action is not taken. Nature Medicine. 2020 May 1;26:819-20.
14. Brar R, Bruneau J, Butt P, Goyer M, Lim R, Poulin G, et al. Medications and other clinical approaches to support physical distancing for people who use substances during the COVID-19 pandemic: National Rapid Guidance Document. Version 1.
Vancouver, BC: Canadian Research Initiative in Substance Misuse; 2020 Jun 22 [Available from: https://crism.ca/wp-content/uploads/2020/06/CRISM-Guidance-Medications-and-other-clinical-approaches-22062020-final.pdf.
15. CCSA. CCENDU Alert: Changes Related to COVID-19 in the Illegal Drug Supply and Access to Services, and Resulting Health Harms: Canadian Centre on Substance Use and Addiction; 2020 [Available from: https://www.ccsa.ca/sites/default/files/2020-05/CCSA-COVID-19-CCENDU-Illegal-Drug-Supply-Alert-2020-en.pdf.
16. Tofighi B, Grossman E, Williams AR, Biary R, Rotrosen J, Lee JD. Outcomes among buprenorphine-naloxone primary care patients after Hurricane Sandy. Addict Sci Clin Pract. 2014;9(1):3.
17. BC Centre for Substance. BC launches 24/7 helpline to help medical professionals care for people with addictions 2020 Jun 16 [Available from: https://www.bccsu.ca/blog/news-release/bc-launches-24-7-helpline-to-help-medical-professionals-care-for-people-with-addictions/.
18. Takeuchi C. As B.C. reports record number of overdose deaths, Dr. Bonnie Henry makes emotional plea for help Online Article: Georgia Straight; 2020 Jun 12 [Available from: https://www.straight.com/living/as-bc-reports-record-number-of-overdose-deaths-dr-bonnie-henry-makes-emotional-plea-for-help.
19. Bains C. Coroner to feds: Take action on B.C. overdose epidemic like with pandemic. Online Article: National Post; 2020 Jun 11 [Available from: https://nationalpost.com/pmn/news-pmn/canada-news-pmn/cp-newsalert-b-c-records-highest-overdose-death-toll-for-one-month-in-may.

20. Shaw R. B.C. boosts funding for substance use outreach teams, amid overdose crisis Online Article: Vancouver Sun; 2020 Jul 14 [Available from: https://vancouversun.com/news/local-news/b-c-boosts-funding-for-substance-use-outreach-teams-amid-overdose-crisis.
21. Samuels EA, Clark SA, Wunsch C, Jordison Keeler LA, Reddy N, Vanjani R, et al. Innovation During COVID-19: Improving Addiction Treatment Access. J Addict Med. 2020;14(4).
22. Galea-Singer S, Newcombe D, Farnsworth-Grodd V, Sheridan J, Adams P, Walker N. Challenges of virtual talking therapies for substance misuse in New Zealand during the COVID-19 pandemic: an opinion piece New Zealand Medical Journal. 2020 May;133(1515).
23. Bruneau J, Rehm J, Wild TC, Wood E, Sako A, Swansburg J, et al. Telemedicine Support for Addiction Services: National Rapid Guidance Document. Version 1. Montreal, Quebec: Canadian Research Initiative in Substance Misuse 2020 May 15 [Available from: https://crism.ca/wp-content/uploads/2020/05/CRISM-National-Rapid-Guidance-Telemedicine-V1.pdf.
24. Rogers BG, Arnold T, Schierberl Scherr A, Strong SH, Holcomb R, Daley Ndoye C, et al. Adapting Substance Use Treatment for HIV Affected Communities During COVID-19: Comparisons Between a Sexually Transmitted Infections (STI) Clinic and a Local Community Based Organization. AIDS Behav. 2020:1-4.
25. Karamouzian M, Johnson C, Kerr T. Public health messaging and harm reduction in the time of COVID-19. Lancet Psychiatry. 2020;7(5):390-1.
